# An increase in integrin-linked kinase non-canonically confers NF-κB-mediated growth advantages to gastric cancer cells by activating ERK1/2

**DOI:** 10.1186/s12964-014-0069-3

**Published:** 2014-11-15

**Authors:** Po-Chun Tseng, Chia-Ling Chen, Yan-Shen Shan, Wen-Teng Chang, Hsiao-Sheng Liu, Tse-Ming Hong, Chia-Yuan Hsieh, Sheng-Hsiang Lin, Chiou-Feng Lin

**Affiliations:** Institute of Clinical Medicine, College of Medicine, National Cheng Kung University, Tainan, 701 Taiwan; Center for Translational Medicine, Taipei Medical University, Taipei, 110 Taiwan; Department of Surgery, College of Medicine, National Cheng Kung University, Tainan, 701 Taiwan; Department of Biological Science and Technology, Chung Hwa University of Medical Technology, Tainan, 717 Taiwan; Department of Microbiology and Immunology, College of Medicine, National Cheng Kung University, Tainan, 701 Taiwan; Center of Infectious Disease and Signaling Research, College of Medicine, National Cheng Kung University, Tainan, 701 Taiwan; Graduate Institute of Medical Sciences, College of Medicine, Taipei Medical University, Taipei, 110 Taiwan; Department of Microbiology and Immunology, College of Medicine, Taipei Medical University, Taipei, 110 Taiwan

**Keywords:** ILK, Cell growth, IQGAP1, ERK1/2, NK-κB

## Abstract

**Background:**

Increased activity or expression of integrin-linked kinase (ILK), which regulates cell adhesion, migration, and proliferation, leads to oncogenesis. We identified the molecular basis for the regulation of ILK and its alternative role in conferring ERK1/2/NF-κB-mediated growth advantages to gastric cancer cells.

**Results:**

Inhibiting ILK with short hairpin RNA or T315, a putative ILK inhibitor, abolished NF-κB-mediated the growth in the human gastric cancer cells AGS, SNU-1, MKN45, and GES-1. ILK stimulated Ras activity to activate the c-Raf/MEK1/2/ERK1/2/ribosomal S6 kinase/inhibitor of κBα/NF-κB signaling by facilitating the formation of the IQ motif-containing GTPase-activating protein 1 (IQGAP1)–Ras complex. Forced enzymatic ILK expression promoted cell growth by facilitating ERK1/2/NF-κB signaling. PI3K activation or decreased PTEN expression prolonged ERK1/2 activation by protecting ILK from proteasome-mediated degradation. C-terminus of heat shock cognate 70 interacting protein, an HSP90-associated E3 ubiquitin ligase, mediated ILK ubiquitination to control PI3K- and HSP90-regulated ILK stabilization and signaling. In addition to cell growth, the identified pathway promoted cell migration and reduced the sensitivity of gastric cancer cells to the anticancer agents 5-fluorouracil and cisplatin. Additionally, exogenous administration of EGF as well as overexpression of EGFR triggered ILK- and IQGAP1-regulated ERK1/2/NF-κB activation, cell growth, and migration.

**Conclusion:**

An increase in ILK non-canonically promotes ERK1/2/NF-κB activation and leads to the growth of gastric cancer cells.

**Electronic supplementary material:**

The online version of this article (doi:10.1186/s12964-014-0069-3) contains supplementary material, which is available to authorized users.

## Background

Integrin-linked kinase (ILK), a 59-kDa serine/threonine kinase, directly interacts with the cytoplasmic domain of β1 integrin [1]. ILK comprises three domains: N-terminal ankyrin (ANK) repeats, a central pleckstrin homology (PH)-like domain, and a C-terminal kinase domain [2,3]. Integrin-mediated cell–extracellular matrix (ECM) adhesion or growth factors activate phosphatidylinositol 3-kinase (PI3K) to phosphorylate membrane-bound PI 4,5-bisphosphate (PIP2) and generate PI 3,4,5-triphosphate (PIP3), which binds to the PH-like domain of ILK and activates ILK [4,5]. After ILK activation, the C-terminal kinase domain of ILK can bind to various proteins, including AKT, affixin, β-parvin, glycogen synthase kinase (GSK)-3β, calponin homology-containing ILK-binding protein, the 20-kDa regulatory light chains of myosin (LC20), the myosin-targeting subunit of myosin light chain phosphatase (MYPT1), paxillin, α-NAC, and the protein phosphatase inhibitors PHI-1, KEPI, and CPI-17 [2,3,6,7]. The N-terminal ANK repeats mediate the interaction of ILK with ILKAP, a protein phosphatase 2C family member, and PINCH, an LIM domain-only adaptor protein. ILK can be considered a PIP3-interacting protein downstream of PI3K; its effects are blocked by phosphatase and tensin homolog deleted on chromosome 10 (PTEN) [8,9]. PTEN suppresses tumors by dephosphorylating PIP3 [10,11].

ILK plays a vital role in regulating various cellular processes, including proliferation, survival, migration, cell cycle progression, and angiogenesis; increased activity or expression of ILK leads to oncogenesis [2,3]. Besides modulating its partner proteins for cellular processes, ILK is hypothesized to be involved in an intracellular signal transduction network. Mechanistically, ILK directly phosphorylates AKT on Ser473 and GSK-3β on Ser9 [4,9] to mediate β-catenin translocation and regulate AP-1 expression for tumor cell proliferation [12]. NF-κB activation is essential for ILK-mediated oncogenic processes, such as anti-apoptotic activity [13], survival promotion [14], epithelial–mesenchymal transition [15], cellular extension and resistance to apoptosis [16], angiogenesis [17], and migration, invasion, and metastasis [18-20]. In addition, NF-κB activation is required for the canonical regulation of IKKα and IKKß by the ILK/AKT pathway. To trigger cell migration, ILK can activate the small GTPases RAC and CDC42 [21]. Furthermore, ILK regulates ERK1/2 activation in myogenic differentiation [22]. Increased expression of microRNA-143 and microRNA-145, which target ILK, inhibits AKT and ERK1/2 pathways [23]. However, the molecular mechanism underlying ILK-mediated ERK1/2 activation remains unknown.

The stimulation of cells by growth factors and cytokines as well as cellular interaction with ECM increase ILK activity [24]. In addition to the molecular regulation of PI3K/PTEN by ILK, Aoyagi et al. identified ILK as a new heat shock protein (HSP) 90 client protein and found that pharmacologically inhibiting HSP90 resulted in ILK degradation in a proteasome-dependent manner [25]. Furthermore, the HSP90-associated E3 ubiquitin ligase C-terminus of heat shock cognate 70 interacting protein (CHIP) causes ILK degradation [26]. Hashiramoto et al. demonstrated that HSP90 stabilized ILK and sustained AKT and ERK1/2 activation [16]. Thus, we speculate a relationship between ILK stability and the activation of its downstream kinases. Ras/MAPK pathway signaling is essential for tumorigenesis [27]. Increased ILK expression is related to high-grade gastric cancer [28], prostate cancer [29], and non-small cell lung cancer [30], although cells in these cancers commonly harbor Ras mutations [31-33]. Targeting ILK with siRNA decreases gastric cancer cell invasion, proliferation, and growth through an unknown mechanism [34]. Regarding the possibility that ILK acts upstream of NF-κB by regulating IKKα [13], which has been implicated in gastric tumorigenesis [35], ILK is speculated to activate cell growth through an NF-κB-regulated pathway. Using gastric cancer cells (AGS, MKN45, and SNU-1), we studied the molecular regulation of ILK and identified a non-canonical pathway of ILK-regulated ERK1/2 activation for NF-κB-mediated gastric cancer cell growth, migration, and survival promotion.

## Results

### ILK activity and expression are essential for NF-κB-mediated cell growth

**Figure 1:**
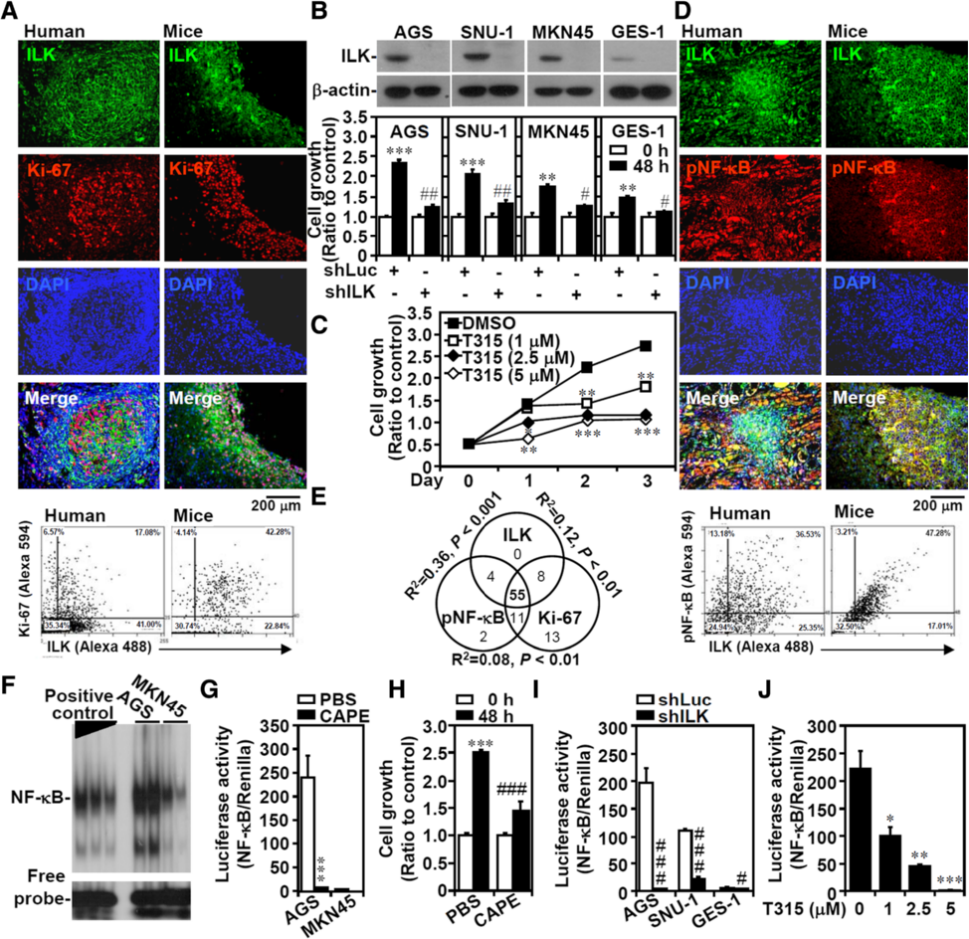
**ILK expression is necessary for cell growth and NF**-**κB activation. (A)** Representative fluorescence-based immunohistochemical staining shows the coexpression of ILK (*green*) and Ki-67 (*red*) in human gastric tumors and AGS-derived nodules in BALB/c mice. DAPI (*blue*) was used for nuclear counterstaining. ILK^+^Ki-67^+^ cells in the immunofluorescently stained tissue sections are presented as dot-plots of a FACS-like analysis by using TissueQuest software. The cells were calculated as the percentage and the cell number of the total cells (DAPI^+^ cells) per field. **(B)** Western blot of ILK expression in cells transfected with shRNAs targeting ILK (*shILK*) or a control luciferase (*shLuc*). β-actin was used as an internal control. WST-8-based assay shows the inhibition of cell growth in ILK-silenced cells. **(C)** The dose-dependent effect of the ILK inhibitor T315 on the inhibition of AGS cell growth. **(D)** Representative fluorescence-based immunohistochemical staining shows the coexpression of ILK (*green*) and phosphorylated NF-κB Ser536 (*red*) in human gastric tumors and AGS-derived nodules in mice. DAPI (*blue*) was used for nuclear counterstaining. The dot-plots show the coexpression of ILK and NF-κB Ser536. **(E)** Logistic regression analysis showed a correlation between ILK, phosphorylated NF-κB Ser536, and Ki-67 in 93 gastric cancer specimens tested. R-squared (*R* ^*2*^) and *P* values are shown. **(F)** EMSA demonstrating NF-κB activation. **(G)** Luciferase reporter assay shows the activation ratio of NF-κB to control Renilla luciferase in cells treated with the NF-κB inhibitor CAPE (25 μg/mL). **(H)** Growth of 25 μg/mL CAPE-treated AGS cells. **(I)** NF-κB activation in shLuc- or shILK-transfected cells. **(J)** NF-κB activation after 6 h of T315 treatment in AGS cells. For cell growth, colony formation, and luciferase activity, data are mean ± SD from three independent experiments. *P <0.05, **P <0.01, and ***P <0.001 compared with Day 0 or relative control. #*P* <0.05, ##*P* <0.01, and ###*P* <0.001 compared with shLuc.

Increased activity or expression of ILK enhances tumorigenesis by promoting cell growth [6]. RNAi-based ILK silencing attenuates gastric cancer cell growth [34], whereas ILK overexpression is related to gastric tumorigenesis [28]. In human gastric tumors and AGS-derived nodules in BALB/c mice, Ki-67-positive proliferating cells coexpressed ILK as demonstrated by the fluorescence-based immunostaining (Figure 1A) and AEC-based immunostaining (Additional file 1: Supplemental materials and methods; Additional file 2: Figure S1) experiments. To investigate the possible mechanisms underlying ILK-mediated gastric cancer cell growth, several gastric epithelial cell lines were characterized according to their different cell growth rates, which were higher for the AGS and SNU-1 cells and lower for the MKN45 and GES-1 cells, and used in this study (Additional file 3: Figure S2A). Compared with the MKN45 cells, the AGS and SNU-1 cells also had elevated ILK expression (Additional file 3: Figure S2B and S2C). A lentiviral-based shRNA was used to silence *ILK* genetically in the AGS, SNU-1, MKN45, and GES-1 gastric epithelial cells (Figure 1B, upper panel) as well as in A549 and H1975 human lung adenocarcinoma cells, HK-2 human renal proximal tubular epithelial cells, and THP-1 human monocytic cells (Additional file 3: Figure S2D). In these cells, ILK silencing significantly (*P* <0.05) decreased cell growth (Figure 1B; Additional file 3: Figure S2E). Furthermore, treating cells with the ILK inhibitor T315 [36] significantly (*P* <0.05) and dose-dependently retarded cell growth (Figure 1C) without cytotoxicity (data not shown). Additionally, decreased colony formation was observed in ILK-silenced AGS cells (Additional file 3: Figure S2F). Thus, gene silencing (Additional file 3: Figure S2G) and pharmacological methods (Additional file 3: Figure S2H) to suppress ILK activity or overexpression led to cell cycle arrest at the G_1_ phase. These results show a growth-promoting role of ILK.

To characterize the features of ILK-regulated cell growth, NF-κB signaling was examined because ILK can act upstream of NF-κB by regulating IKKα [13]. By immunostaining, the coexpression of ILK and phosphorylated NF-κB (Ser536) was observed in human and mouse gastric tissues (Figure 1D), and their coexpression significantly (*P* <0.01) and positively correlated with the number of proliferating cells, which is indicated by 55 triple-positive cases of the total 93 gastric cancer specimens (Figure 1E; Additional file 4: Figure S3). Immunostaining for NF-κB nuclear translocation (Additional file 3: Figure S2I), EMSA (Figure 1F), and promoter assays (Figure 1G) confirmed the constitutive activation of NF-κB in the AGS cells but not in the MKN45 cells. Treating cells with the NF-κB inhibitor CAPE significantly (*P* <0.001) reduced NF-κB activation (Figure 1G) and cell growth (Figure 1H). Either ILK silencing (Figure 1I; Additional file 3: Figure S2J) or T315 treatment (Figure 1J) significantly (*P* <0.05) stopped NF-κB activity. These results demonstrated that ILK is indispensable for cell growth in the cell lines tested because it facilitates NF-κB activation in gastric cancers.

### ILK regulates Ras activity by facilitating the complex of IQGAP1–Ras to control MAPK-activated NF-κB

**Figure 2:**
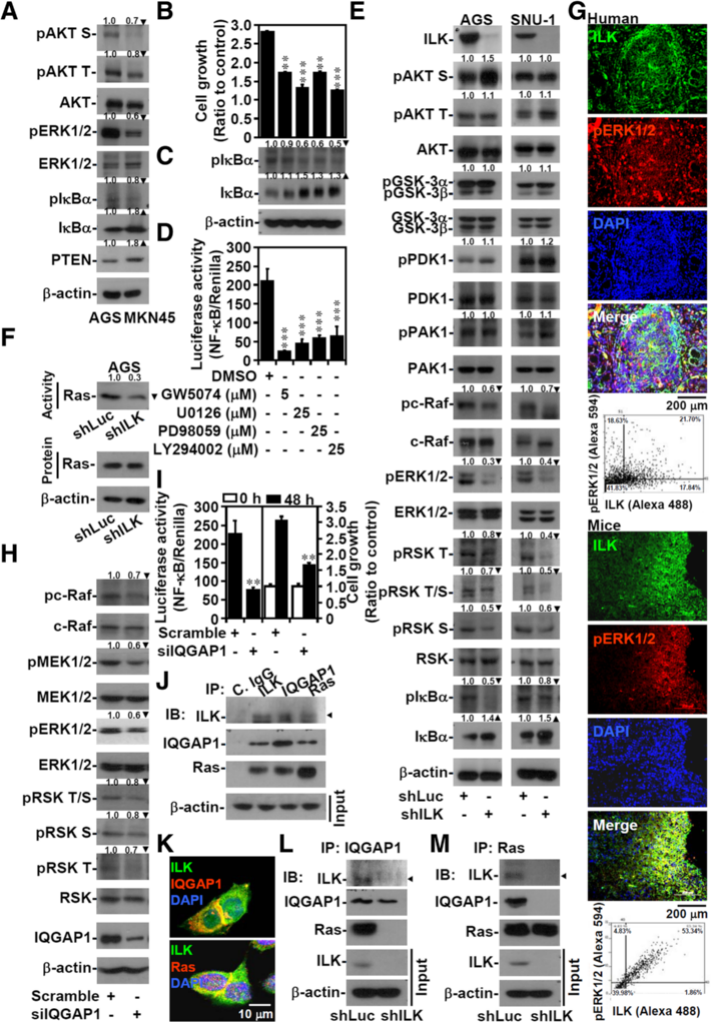
**ILK**–**IQGAP1**–**Ras complex sustains Ras activity to activate c**-**Raf**/**MEK1**/**2**/**ERK1**/**2**/**RSK**/**IκBα**/**NF**-**κB signaling. (A)** Western blot of the indicated proteins in AGS and MKN45 cells. β-actin was used as an internal control. AGS cells treated with c-Raf inhibitor GW5074, MEK1/2 inhibitors U0126 or PD98059, or PI3K inhibitor LY294002 for 48 h were evaluated for their growth **(B)**, phosphorylation of IκBα at Ser32 (*pIκBα*) and total protein **(C)**, and NF-κB activation **(D)**. **(E)** In shLuc- or shILK-transfected cells, western blots show the expression of the indicated proteins. β-actin was used as an internal control. **(F)** Ras activation assay showing Ras activity and protein expression in shLuc- and shILK-transfected AGS cells. **(G)** Representative fluorescence-based immunohistochemical staining showing the coexpression of ILK (*green*) and phosphorylated ERK1/2 Tyr202/Thr204 (*red*) in human gastric tumors and AGS-derived nodules in BALB/c mice. DAPI (*blue*) was used for nuclear counterstaining. The dot-plots show the coexpression of the indicated proteins. In control and IQGAP1 siRNA-transfected AGS cells at 48 h, the expression of the indicated proteins **(H)**, NF-κB activation, and cell growth **(I)** were shown. Protein lysates extracted from untreated AGS cells **(J)** or with shLuc and shILK transfection **(L, M)** were immunoprecipitated (*IP*) with control IgG (*C. IgG*) or with antibodies against ILK, IQGAP1, and Ras. Immunoblots (*IB*) show the expression of ILK, IQGAP1, and Ras. **(K)** Representative fluorescence-based immunohistochemical staining showing the coexpression of ILK (*green*), IQGAP1 (*red*), and Ras (*red*) in AGS cells. DAPI (*blue*) was used for nuclear counterstaining. For proliferation and luciferase activity, data are mean ± SD from three independent experiments. **P <0.01 and ***P <0.001 compared with control. Upright triangle, increased expression; inverted triangle, decreased expression.

Because AGS cells harbor *PIK3CA* and *KRAS* mutations [37], we examined possible regulatory effects of ILK on the modulation of NF-κB activity by these 2 kinases [38]. Using a Human Phospho-MAPK Array Kit, we identified 10 kinases that were more highly expressed in the AGS cells than in the MKN45 cells. These kinases mostly acted downstream of the PI3K and MAPK signaling pathways (Additional file 5: Figure S4A). By western blotting, we confirmed an increased phosphorylation of AKT, ERK1/2, and IκBα accompanied by IκBα degradation in the AGS cells (Figure 2A). The pharmacological inhibition of c-Raf, MEK1/2, and PI3K significantly (*P* <0.05) reduced cell growth (Figure 2B), IκBα phosphorylation (Ser32) and degradation (Figure 2C), and NF-κB activity (Figure 2D), indicating that both PI3K- and Ras-activating signaling pathways facilitated NF-κB activation. The effects of ILK have been widely studied because of its interactions with cell growth- and NF-κB-associated AKT [4,9]. Surprisingly, ILK silencing did not affect AKT and GSK-3β phosphorylation in the AGS and SNU-1 cells but markedly reduced c-Raf and ERK1/2 activation in all cells tested (Figure 2E; Additional file 5: Figure S4B). Without AKT deactivation, we evaluated an alternative pathway for activating NF-κB through a mechanism involving MAPK/p90RSK/IκBα signaling [38]. The knockdown of *ILK* reduced the multiple phosphorylation of RSK (Thr573, Thr359/Ser363, and Ser380) and IκBα phosphorylation (Ser32) and increased IκBα accumulation (Figure 2E). Inhibiting MEK1/2 caused similar effects (Additional file 5: Figure S4C) and a cell cycle arrest at the G_1_ phase (Additional file 5: Figure S4D). A Ras pull-down assay revealed that inhibiting ILK caused Ras deactivation without affecting the stability of the Ras protein (Figure 2F). These findings demonstrated a potential non-canonical pathway for ILK to modulate NF-κB by regulating Ras/c-Raf/MEK1/2/ERK1/2/IκBα signaling.

ILK can modify ERK1/2 activation under cell growth and differentiation [22]; however, the molecular regulation related to Ras signaling has not been documented [2,3]. The coexpression of ILK and phosphorylated ERK1/2 (Tyr202/Thr204) was demonstrated in human gastric tumors and AGS-derived nodules in BALB/c mice (Figure 2G). ILK interacts with IQGAP1 [7], a Ras GTPase-activating-like protein that is dissimilar from GAP, which transforms Ras to its inactive state. Because IQGAP1 controls Ras/MAPK signaling, it is oncogenic [39,40]. The expression of IQGAP family proteins was unchanged in the AGS and MKN45 cells, even after ILK silencing (Additional file 5: Figure S4E). However, silencing oncogenic IQGAP1, but not IQGAP3, (Additional file 5: Figure S4F–S4H) effectively inhibited c-Raf/MEK1/2/ERK1/2/RSK signaling (Figure 2H), NF-κB activity, and cell growth (Figure 2I). By performing a coimmunoprecipitation assay, we demonstrated a potential complex harboring ILK, IQGAP1, and Ras (Figure 2J). Immunostaining confirmed that ILK was coexpressed at levels similar to those of IQGAP1 and Ras (Figure 2K; Additional file 6: Figure S5). Notably, silencing ILK disrupted the IQGAP1–Ras complex (Figure 2L and 2M). These results demonstrated that ILK facilitated the formation of the IQGAP1–Ras complex to sustain Ras activity.

### Enzymatic ILK modulates the formation of the IQGAP1–Ras complex and ERK1/2-mediated cell growth

**Figure 3:**
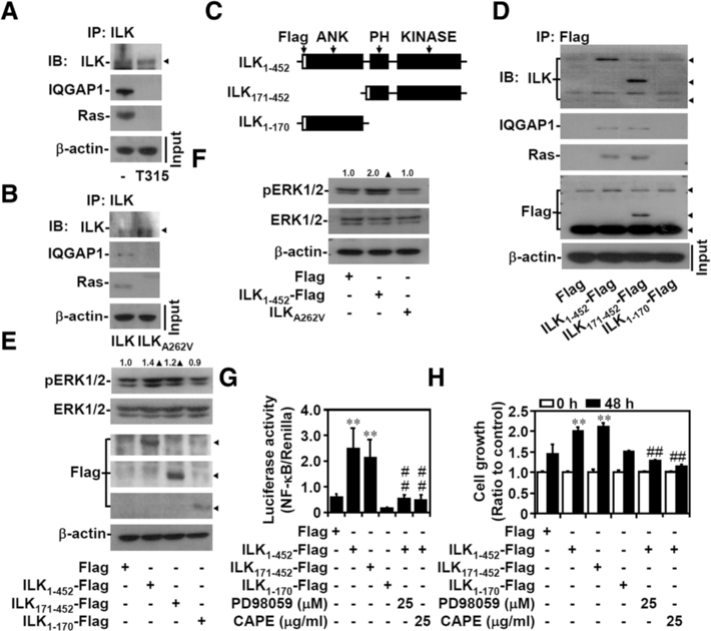
**Enforced ILK expression facilitates cell growth by inducing ERK1**/**2**/**NF**-**κB activation**. Protein lysates extracted from AGS cells treated with T315 **(A)** or transfected with Myc-DDK-tagged mutant ILK_A262V_ **(B)** were immunoprecipitated (*IP*) with antibodies against ILK. Immunoblots (*IB*) show the expression of ILK, IQGAP1, and Ras. Plasmids based on pcDNA3.1-Flag-ILK containing full-length ILK (*ILK*_*1*–*452*_), fragment 171–452 (*ILK*_*171*–*452*_), fragment 1–170 (*ILK*_*1*–*170*_) **(C)**, or Myc-DDK-tagged mutant ILK_A262V_ were transfected into MKN45 cells. **(D)** Coimmunoprecipitation of Flag with the indicated proteins is shown by using western blotting. Compared with pcDNA3.1-Flag (*Flag*) only, western blots show the expression of phosphorylated ERK1/2 at Tyr202/Thr204 (*pERK1*/*2*) **(E, F)**. β-actin was used as an internal control. NF-κB activation **(G)** and cell growth **(H)** were also detected in the absence or presence of PD98059 and CAPE. Data are mean ± SD from three independent experiments. **P <0.01 compared with control. ##*P* <0.01 compared with ILK_1–452_. Upright triangle, increased expression.

Our findings showed that pharmacologically inhibiting ILK decreased cell growth, indicating the essential role of the enzymatic activity of ILK. Further inhibiting ILK by the genetic approach disrupted IQGAP1-mediated Ras signaling. Inhibiting ILK activity pharmacologically with T315 (Figure 3A) or genetically by transfecting the enzymatic mutant ILK_A262V_ [41] (Figure 3B) also disrupted the formation of the IQGAP1–Ras complex in the AGS cells. Three truncated regions of ILK (Figure 3C) were overexpressed in the MKN45 cells to identify the domain essential for sustaining the IQGAP1–Ras complex. Coimmunoprecipitation assays demonstrated that overexpressed ILK constructs harboring PH and catalytic kinase domain regions immunoprecipitated with IQGAP1 and Ras (Figure 3D). Therefore, only kinase domain-containing ILK activated ERK1/2 (Figure 3E). Compared with the full-length ILK (ILK_1–452_), transfecting the kinase-dead mutant ILK_A262V_ did not activate ERK1/2 (Figure 3F). Additional experiments demonstrated that only kinase domain-containing ILK increased MEK1/2-regulated NF-κB activation (Figure 3G) followed by MEK1/2- and NF-κB-regulated cell growth (Figure 3H) in the MKN45 cells. These results showed that enzymatic ILK mediated the formation of the IQGAP1–Ras complex to trigger ERK1/2- and NF-κB-mediated cell growth.

### PI3K activation and decreased PTEN expression facilitate ERK1/2/NF-κB activation by stabilizing ILK

**Figure 4:**
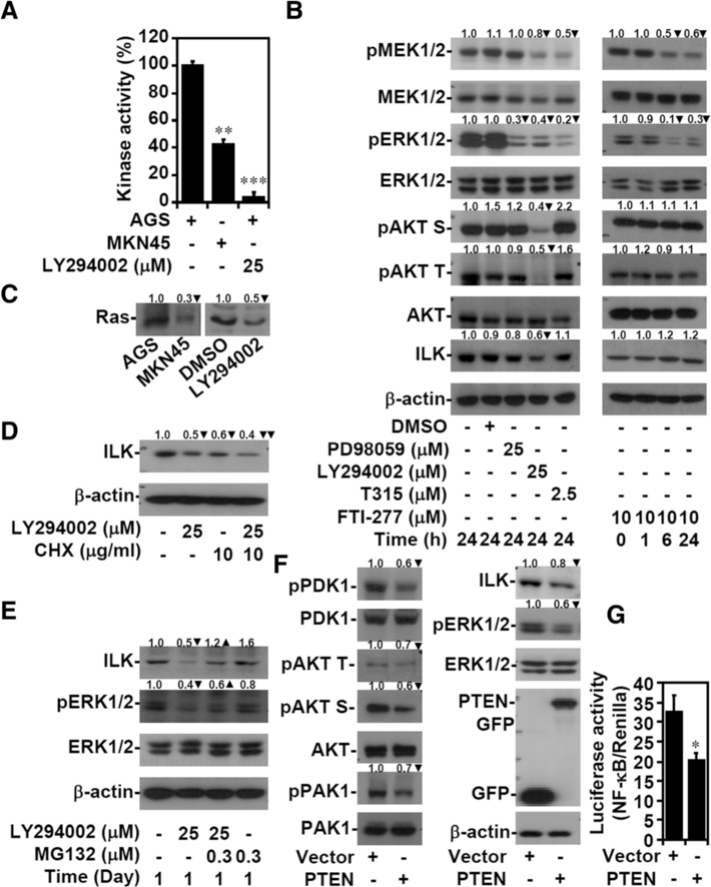
**PI3K activation and decreased PTEN expression facilitate ERK1**/**2**/**NF**-**κB activation through ILK stabilization. (A)** A PIP3 Mass ELISA Kit was used to determine PI3K activity by measuring the amount of PIP3 extracted from untreated AGS and MKN45 cells and from AGS cells treated with LY294002 for 24 h. **(B)** AGS cells were treated with PD98059, LY294002, T315, or Ras inhibitor FTI-277 for the indicated times. Western blots show the expression of the indicated proteins. β-actin was used as an internal control. **(C)** Ras activity was detected in AGS, MKN45, and LY294002-treated AGS cells after 24 h. Western blots show the expression of the indicated proteins in AGS cells pretreated with the translation inhibitor cycloheximide (*CHX*) **(D)** or proteasome inhibitor MG132 **(E)** for 0.5 h and then treated with LY294002 for 24 h. PTEN was overexpressed in AGS cells by using pcDNA3.1-GFP-PTEN (*PTEN*). Western blots show the expression of the indicated proteins compared with pcDNA3.1-GFP (*Vector*) transfection **(F)**, and NF-κB activation **(G)** was also determined. For kinase and luciferase activity, data are mean ± SD from three independent experiments. *P <0.05, **P <0.01, and ***P <0.001 compared with control. Upright triangle, increased expression; inverted triangle, decreased expression.

Because ILK is dependent on PI3K-mediated PIP3 generation for its activation [4], growth factor- and integrin-mediated PI3K activation or a PI3K mutation in the AGS cells [37] could contribute to ILK expression and activation. Compared with the growth of the MKN45 cells, the growth of the AGS cells was unaffected by elevated IL-6 levels [42], and the expression of β1 and β3 integrins did not increase (Additional file 7: Figure S6). We further confirmed that PIP3 generation was higher in the PI3K-mutated AGS cells (Figure 4A). To explore the relationships among the PI3K, ILK, and Ras signaling pathways, the AGS cells were treated with the MEK1/2, PI3K, ILK, or Ras inhibitor for different durations. At 6 h after treatment, T315 slightly inhibited MEK1/2 and ERK1/2 phosphorylation (Additional file 8: Figure S7). At 24 h after treatment, PI3K inhibition disrupted ILK expression, which was followed by MEK1/2/ERK1/2 deactivation 24 h after treatment (Figure 4B). However, Ras inhibition did not deactivate AKT or ILK in the AGS cells, although Ras can mediate PI3K activation [27]. Similar to the ILK-silenced AGS cells, both MKN45 and LY294002-stimulated AGS cells showed reduced Ras activity (Figure 4C). The translation inhibitor cycloheximide facilitated LY294002-induced ILK downregulation (Figure 4D) and the proteasome inhibitor MG132 reversed the aforementioned effect and ERK1/2 inactivation (Figure 4E). The tumor suppressor phosphatase PTEN negatively regulates PI3K/PDK1/AKT signaling [10,11] and ILK activity [8,9], and the AGS cells exhibited a low expression of PTEN (Figure 2A) [43]. The forced expression of PTEN in the AGS cells not only attenuated the constitutive phosphorylation of PDK1, AKT, and PAK1 but also inhibited ILK expression, ERK1/2 phosphorylation (Figure 4F), and NF-κB activation (Figure 4G). These results indicated that PI3K activation and decreased PTEN expression stabilize ILK to regulate ERK1/2/NF-κB activation.

### HSP90-associated E3 ligase CHIP negatively controls ILK stabilization, ERK1/2/NF-κB activation, and cell growth

**Figure 5:**
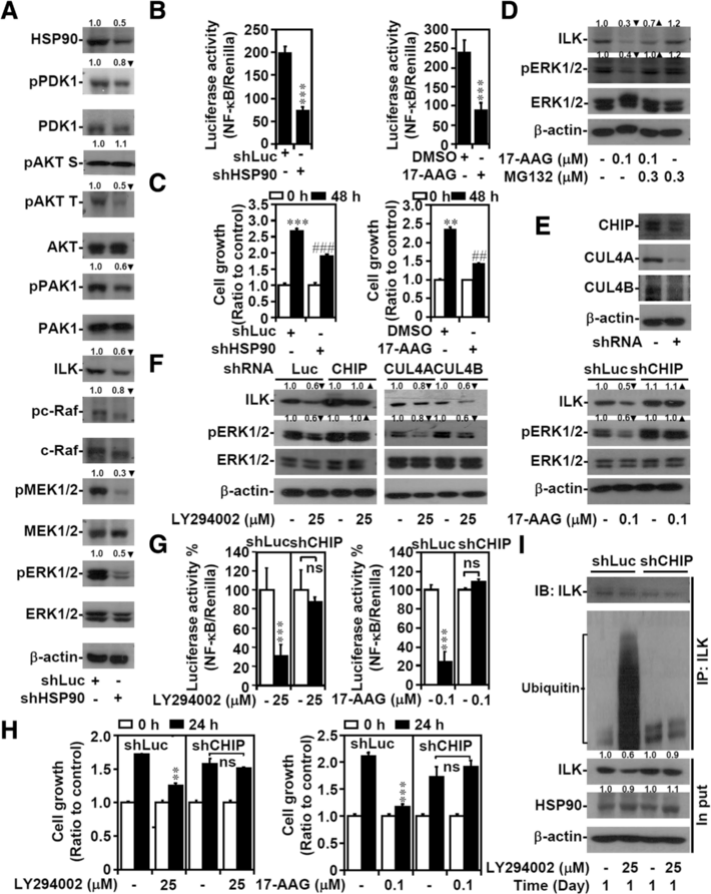
**CHIP determines ILK destabilization**, **ERK1**/**2**/**NF**-**κB inactivation**, **and cell growth inhibition after PI3K**/**HSP90 inactivation**. AGS cells were transfected with shLuc or shHSP90 or pretreated with the HSP90 inhibitor 17-AAG (0.1 μM) for 48 h. Western blots show the expression of the indicated proteins **(A)**, and NF-κB activation **(B)** and cell growth **(C)** were also determined. **(D)** MG132 pretreatment (0.5 h) reversed ILK expression and phosphorylated ERK1/2 at Tyr202/Thr204 (*pERK1*/*2*) in 17-AAG-treated AGS cells. **(E)** CHIP, cullin 4A, and cullin 4B were silenced in AGS cells by shRNAs. shRNA-transfected cells were treated with LY294002 or 17-AAG for 24 h. Western blotting showed the expression of ILK and phosphorylated ERK1/2 at Tyr202/Thr204 (*pERK1*/*2*) **(F)**, and NF-κB activation **(G)** and cell growth **(H)** were also determined. **(I)** In shLuc- and shCHIP-transfected AGS cells treated with LY294002, ILK was immunoprecipitated, and its ubiquitylation was probed. For western blot analysis, β-actin was used as an internal control. For luciferase activity and cell growth, data are mean ± SD from three independent experiments. **P <0.01 and ***P <0.001 compared with control. ##*P* <0.01 and ###*P* <0.001 compared with shLuc or DMSO. NS: not significant. Upright triangle, increased expression; inverted triangle, decreased expression.

By further verifying the mechanism underlying ILK destabilization, our results showed that AKT inhibition did not affect ILK stability, whereas PI3K inhibition affected ILK stability (Additional file 9: Figure S8). HSP90 was next investigated because it can cause ILK degradation [25]. Similar to the results of PI3K inhibition, shRNA-based (Figure 5A) or pharmacological inhibition of HSP90 (Additional file 10: Figure S9A) delayed not only PDK1/AKT phosphorylation but also c-Raf/MEK1/2/ERK1/2 activation after ILK degradation. Furthermore, genetically or pharmacologically inhibiting HSP90 by treatment with 17-AAG or the HDAC inhibitor SAHA, which traps HSP90 in an acetylated, enzymatically inactive state, attenuated NF-κB activity (Figure 5B; Additional file 10: Figure S9B) and cell growth (Figure 5C). Cotreatment with MG132 rescued the effects of 17-AAG (Figure 5D) and SAHA on ILK degradation and ERK1/2 inactivation (Additional file 10: Figure S9C). In the MKN45 cells, treatment with MG132 increased ILK expression, MEK1/2 and ERK1/2 phosphorylation (Additional file 11: Figure S10), and NF-κB activation (data not shown). Coimmunoprecipitation demonstrated the formation of the HSP90–ILK complex (data not shown). Using a lentiviral-based shRNA approach (Figure 5E), HSP90-associated E3 ligases, including CHIP, cullin 4A, and cullin 4B [26,44,45], were examined for their roles in ILK degradation. The results showed that only silencing CHIP rescued ILK degradation and ERK1/2 inactivation (Figure 5F), NF-κB deactivation (Figure 5G), and cell growth inhibition (Figure 5H) after the pharmacological inhibition of PI3K and HSP90. CHIP was required for ILK ubiquitination in the LY294002-treated AGS cells (Figure 5I). These results demonstrated that HSP90/CHIP signaling regulates PI3K-mediated ILK stabilization and ILK-regulated ERK1/2/NF-κB activation and thus facilitates cell growth.

### PI3K/HSP90/CHIP/ILK/IQGAP1/ERK1/2/NF-κB signaling contributes to cell migration and sensitivity to 5-FU and cisplatin

**Figure 6:**
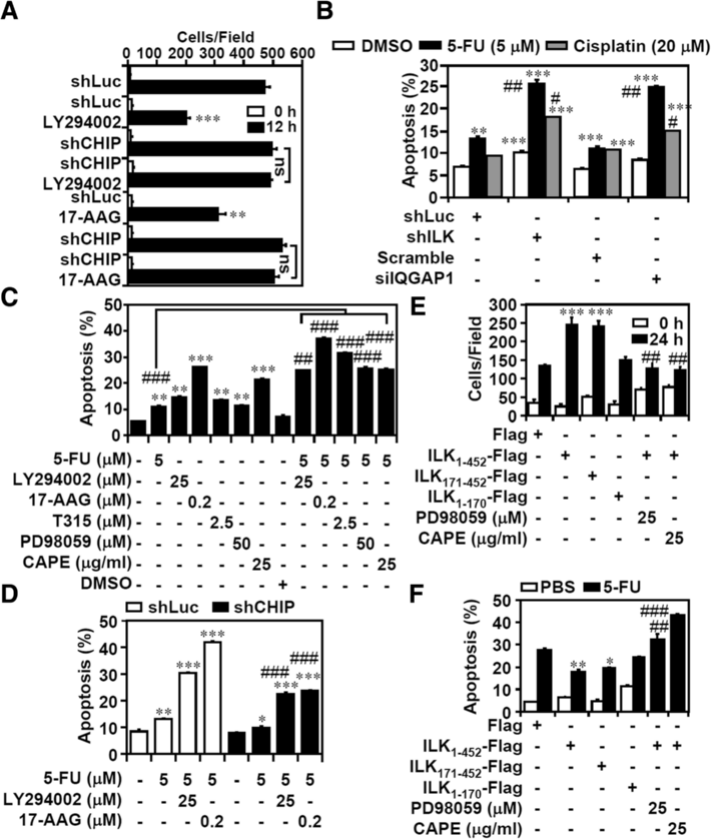
**PI3K**/**HSP90**/**CHIP**/**ILK**/**ERK1**/**2**/**NF**-**κB signaling determines cell migration and controls susceptibility to the anticancer agents 5**-**FU and cisplatin. (A)** Migration activity was tested in shLuc- or shCHIP-transfected AGS cells treated with LY294002 (25 μM) and 17-AAG (0.2 μM) for 12 h. shLuc, control siRNA, or DMSO were used as negative controls. The numbers of migrated cells in the observed field are shown. PI staining followed by flow cytometry analysis showed 5-FU- or cisplatin-induced apoptosis for 2 days in shILK- or siIQGAP1-transfected AGS cells **(B)**, AGS cells pretreated with the indicated inhibitors for 0.5 h **(C)**, and shLuc- or shCHIP-transfected AGS cells treated with LY294002 or 17-AAG **(D)**. shLuc, control siRNA, or DMSO were used as negative controls. Plasmids based on pcDNA3.1-Flag-ILK containing full-length ILK (*ILK*_*1*–*452*_), fragment 171–452 (*ILK*_*171*–*452*_), or fragment 1–170 (*ILK*_*1*–*170*_) were transfected into MKN45 cells. Compared with pcDNA3.1-Flag (*Flag*) only, cell migration **(E)** and apoptosis induced by 5-FU (5 μM) **(F)** were detected in the absence or presence of PD98059 and CAPE. Data are mean ± SD from three independent experiments. *P <0.05, **P <0.01 and ***P <0.001 compared with control. #*P* <0.05, ##*P* <0.01, and ###*P* <0.001 compared with control. NS: not significant.

Increased ILK activity or expression could regulate oncogenic processes, including cell proliferation, migration, and survival [3,6]. We studied the potential involvement of the identified PI3K/HSP90/CHIP/ILK/IQGAP1/ERK1/2/NF-κB signaling pathway in cell growth advantages. Compared with the MKN45 cells, the AGS cells showed increased migratory activity; however, ILK or IQGAP1 silencing significantly (*P* <0.001) abolished cell migration (Additional file 12: Figure S11A). Pharmacologically blocking PI3K/HSP90/ILK/MEK1/2/NF-κB signaling in the AGS cells also significantly (*P* <0.001) attenuated the migratory ability (Additional file 12: Figure S11B), and CHIP knockdown considerably reversed the inhibitory effects caused by the inhibition of PI3K and HSP90 (Figure 6A; Additional file 12: Figure S11C). The anticancer agents 5-FU and cisplatin are commonly used in the treatment of gastric cancers [46]. Treating the AGS cells with 5-FU or cisplatin, particularly after ILK or IQGAP1 silencing, caused increased susceptibility to apoptosis (Figure 6B). T315 increased the sensitivity of the AGS cells to 5-FU and cisplatin (Additional file 12: Figure S11D). Pharmacologically inhibiting PI3K/HSP90/ILK/MEK1/2/NF-κB signaling also sensitized the AGS cells to 5-FU-induced apoptosis (Figure 6C), whereas CHIP silencing retarded the synergistic effects of LY294002 and 17-AAG on 5-FU-induced apoptosis (Figure 6D). In contrast, the overexpression of ILK containing a catalytic kinase domain, including ILK_1–452_ and ILK_171–452_, enhanced MEK1/2/NF-κB-regulated cell migration (Figure 6E) and cell survival after treatment with 5-FU (Figure 6F). These results indicated the common effects of ILK and IQGAP1 on ERK1/2/NF-κB activation for cell migration and survival.

### EGFR signaling regulates ILK/IQGAP1 to activate ERK1/2, NF-κB, and cell migration and proliferation

**Figure 7:**
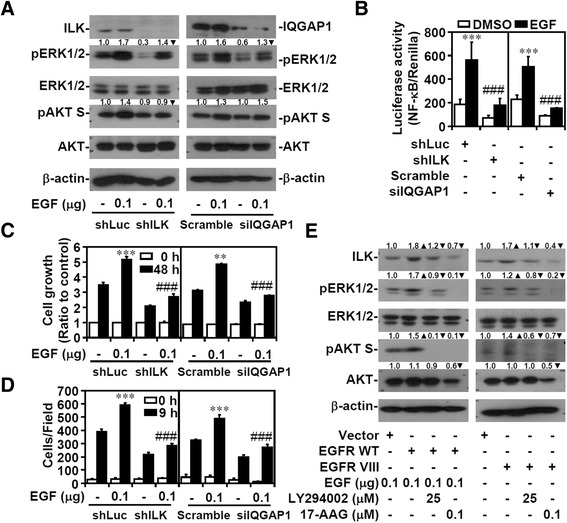
**ILK and IQGAP1 facilitate EGF**/**EGFR**-**induced NF**-**κB activation and cell growth and migration**, **and activated EGFR increases ILK expression. (A)** Western blots showing ERK1/2 phosphorylation at Tyr202/Thr204 (*pERK1*/*2*) and AKT phosphorylation at Ser473 (*pAKT S*) in shILK- or siIQGAP1-transfected AGS cells treated with EGF for 1 h. shLuc and control siRNA were used as negative controls. β-actin was used as an internal control. Furthermore, in these cells, NF-κB activation **(B)**, cell growth **(C)**, and migration **(D)** were detected. Data are mean ± SD from three independent experiments. **P <0.01 and ***P <0.001 compared with negative control. ###*P* <0.001 compared with shLuc or control siRNA. **(E)** In EGFR WT-transfected and EGFR VIII-transfected MKN45 cells treated with or without EGF for 1 h plus LY294002 or 17-AAG 24 h posttreatment, western blots show the expression of ILK, phosphorylated ERK1/2 at Tyr202/Thr204 (*pERK1*/*2*), and phosphorylated AKT at Ser473 (*pAKT S*). β-actin was used as an internal control. Upright triangle, increased expression; inverted triangle, decreased expression.

To verify the role of ILK/IQGAP1-mediated ERK1/2 and NF-κB activation, we examined EGF/EGFR signaling as previously described [47,48]. ILK or IQGAP1 silencing partially eliminated the exogenous EGF-induced ERK1/2 and AKT phosphorylation in the AGS (Figure 7A) and A549 cells (Additional file 13: Figure S12A). However, IQGAP1 silencing attenuated EGF-activated ERK1/2 but not EGF-activated AKT, suggesting an IQGAP1-independent AKT activation. ERK1/2 phosphorylation through multiple stimuli, including hydrogen peroxide (Additional file 13: Figure S12B) and *Helicobacter pylori* infection (Additional file 13: Figure S12C) [49-51], was also abolished in the ILK-silenced cells. Silencing ILK or IQGAP1 caused a significant (*P* <0.001) decrease in EGF-induced NF-κB activation (Figure 7B), and cell growth (Figure 7C) and migration (Figure 7D). The wild-type and the constitutively active form of EGFR mutated at domain VIII [48,52] were transfected into the MKN45 cells, which were then treated with or without EGF. ILK overexpression was accompanied by ERK1/2 and AKT activation; however, inhibiting PI3K and HSP90 decreased not only ILK expression but also ERK1/2 and AKT activation (Figure 7E). These findings demonstrated that both ILK and IQGAP1 are required for EGF/EGFR-triggered ERK1/2/NF-κB activation and cell growth advantages and that ILK expression can be positively regulated by EGF/EGFR/PI3K/HSP90 signaling.

## Discussion

**Figure 8:**
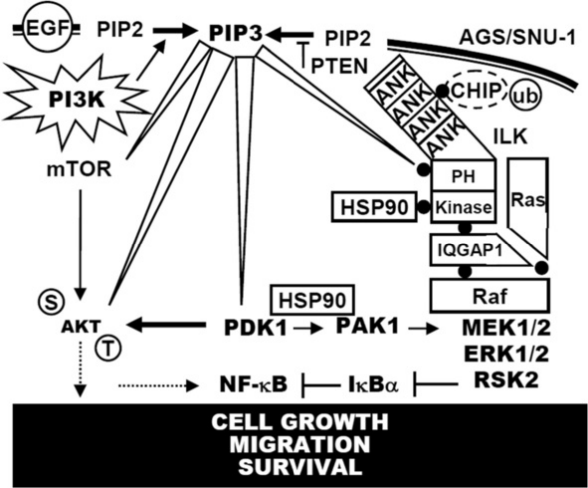
**A hypothetical model for PI3K**/**HSP90**-**regulated ILK**, **followed by ILK**-**mediated IQGAP1**/**Ras**/**c**-**Raf**/**MEK1**/**2**/**ERK1**/**2**/**RSK**/**NF**-**κB activation in cell growth**, **migration**, **and survival**.

By using genetic and pharmacological approaches, we confirmed the proliferation-promoting role of ILK in vitro in gastric cancer cells. The function of ILK is highly related to NF-κB activation. An in vivo model of gastric cancer in mice showed the essential role of ILK in tumor growth [34], and increased ILK [28] and NF-κB [35] activity or expression is related to gastric tumorigenesis. Although the potent mechanisms for ILK-regulated cell proliferation have been previously documented [6], we further investigated the molecular basis of ILK-mediated gastric cancer cell growth, which is related to NF-κB activation. To the best of our knowledge, clinical observations in this study indicated a positive correlation between ILK expression, NF-κB activation, and cell proliferation in gastric cancers. Protection from apoptosis [13] and the stimulation of cell proliferation are oncogenic effects of ILK that are generally achieved by facilitating NF-κB activation. Based on these rationales, the molecular regulation of the ILK/NF-κB pathway was examined. According to the present study results (summarized in Figure 8), increased ILK activity or expression, which is controlled by PI3K/HSP90-mediated protein stabilization, triggers a non-canonical pathway of IQGAP/Ras/c-Raf/MEK1/2/ERK1/2/RSK/NF-κB signaling to stimulate cell growth, migration, and survival.

Aberrant ILK is involved in tumorigenesis, and it is speculated that ILK canonically stimulates AKT to promote NF-κB-mediated oncogenic processes, such as anti-apoptosis [13] and survival [14]. In tumorigenesis, NF-κB can promote survival and proliferation, angiogenesis, adhesion/invasion/metastasis, and inflammation. However, the mechanisms underlying NF-κB activation in cancer cells are multifaceted; both AKT and MAPKs are crucial for NF-κB activation [38]. Of note, AGS cells have mutated *PIK3CA* and *KRAS* [37]. Inhibiting PI3K and Ras activity attenuated NF-κB activation. We therefore hypothesized a role for ILK to canonically or non-canonically act upstream of AKT and ERK1/2, respectively. ILK-mediated AKT phosphorylation at Ser473 was confirmed in lung adenocarcinoma A549 cells but not in gastric cancer cells. Surprisingly, ILK silencing effectively negatively affected ERK1/2 activation in all cells tested. In addition to the kinase activity of ILK, scaffold functions of ILK associated with intracellular molecules also need further investigation to verify its roles in regulating AKT and ERK1/2 signaling. ERK1/2 mediates NF-κB activation through a mechanism involving MAPK/p90RSK/IκBα signaling [38]; similarly, we demonstrated that this pathway was required for ILK-mediated NF-κB activation in gastric cancer cells. ILK has a possible upstream role in ERK1/2 activation [16,22,23,48], but no rational mechanisms exist for examining this regulation [2,3].

An emerging and widely demonstrated role for IQGAP1 is its control over diverse biological functions by interacting with various cellular factors [53]. For MAPK signaling, IQGAP1 can directly regulate Ras/ERK1/2 activation during tumorigenesis [39,40]. Ras is a small GTPase that can hydrolyze GTP into GDP in a GTP-bound protein to inactivate the protein, a process accelerated by GAPs, and this process can be reversed with a guanine nucleotide exchange factor to reactivate the Raf/MAPK pathway [27]. IQGAP is a Rho–GTP-binding protein and has a region similar to that of Ras GAP. However, IQGAP has no GAP function but can stabilize the GTP-bound protein in an activated state [54,55]. A global analysis of the ILK interactome has shown a strong ILK–IQGAP1 interaction [7]; however, no studies have illustrated the axis of ILK/IQGAP1/Ras signaling. Wickstrom et al. demonstrated an ILK–IQGAP1 association through the non-ANK repeats of ILK and the IQ motif of IQGAP1 for facilitating integrin signaling [56]. To our knowledge, the present study is the first to show that increased or decreased activity or expression of ILK did not affect IQGAP1 expression, although silencing ILK disrupted the IQGAP1–Ras interaction. These three proteins formed a novel complex around the cell membrane, particularly in cell–cell junctions. Regarding IQGAP1 sustaining Ras activity in gastric cancer cells, IQGAP1 but not IQGAP3 mediated cell growth through an ERK1/2-regulated NF-κB activation pathway. The present study reveals a novel axis of ILK/IQGAP1 for Ras/ERK1/2 signaling; however its cellular significance requires further investigation. The abnormal expression of IQGAP1 is related to poor prognosis in gastric cancers [57]. The formation of an ILK–IQGAP1 complex may have multiple biological effects; IQGAP1 has been implicated in diverse cellular functions through a mechanism involving the interaction of cytoskeletal components, small GTPases, kinases, and receptors [53].

Besides using the genetic approach, pharmacologically inhibiting ILK with T315 or an ILK kinase-dead mutation (ILK_A262V_) abolished the IQGAP1–Ras interaction and thus deactivated ERK1/2. The enzymatic activity of ILK is speculated to be crucial for the axis of ILK/IQGAP1/Ras signaling. Further results showed the importance of the PH-like domain and the kinase domain in mediating the formation of ILK/IQGAP1 complex as well as ERK1/2 activation, NF-κB activation, and cell growth. The mechanism underlying the ILK-mediated IQGAP1–Ras interaction is unknown; ILK phosphorylates its substrates as well as acts as an adaptor for protein–protein interactions [2,3]. Together with the findings that the non-ANK repeats of ILK are required for binding with the IQ domain of IQGAP1 [53,56], it is reasonable to hypothesize that ILK initiates the phosphorylation of the IQGAP1–Ras complex and/or that ILK confers an adaptor-mediated interaction for this complex.

Although increased ILK expression contributes to tumorigenesis, the mechanisms for ILK overexpression remain unknown. ILK upregulation and activation are related to β1 integrin signaling [1], and integrins may regulate gastric tumorigenesis, particularly by modulating adhesion and metastasis [58,59]. Thus, integrin overexpression causes ILK upregulation and activation. However, in the gastric cancer cell lines AGS and MKN45 that we tested, there were no differences in the expression or activation of β1 or β3 integrin. In addition to integrin signaling, growth factors generally activate PI3K for cell growth advantages. The expression of cell growth-associated IL-6 is higher in the AGS cells than in the MKN45 cells; however, its potential role in gastric cancer cell proliferation has been excluded. In addition to *PIK3CA* mutations [37], growth factors involved in regulating ILK expression need further investigation.

Surprisingly, pharmacologically inhibiting PI3K decreased ILK expression along with ERK1/2 deactivation. This result indicates an upstream role for PI3K-regulated ILK stabilization linked to Ras/ERK1/2 signaling. In the AGS cells, ILK overexpression may result from a natural mutation in *PIK3CA* that generates PIP3 to stabilize ILK expression. The most important finding in this study is the mechanism of ILK-dependent ERK1/2 activation demonstrated in the AGS cells, although these cells harbor *PI3CA* and *KRAS* mutations [37]. ILK may be required to sustain Ras activity, although Ras is automatically activated. The PH-like domain is essential for ILK to interact with PIP3 [4]. Once PI3K-driven PIP3 generation mediates ILK recruitment, ILK signaling can be initiated after protein stabilization. It is speculated that aberrant PI3K activation can cause ILK upregulation. The level of PTEN, a tumor suppressor related to PIP3 downregulation, is markedly decreased in several cancers, including gastric cancers. Restoring PTEN expression in the AGS cells effectively abolished both AKT and ERK activation and decreased ILK expression. PI3K/PTEN/PIP3-mediated ILK stabilization is therefore important for ERK1/2 activation.

PDK1 activates PAK1 [60], and PAK1 phosphorylates ILK [61]. Here, the pharmacological inhibition of PI3K inhibited PDK1 and PAK1 activation (Additional file 14: Figure S13A); however, PAK1 knockdown did not alter ILK expression but decreased c-Raf/ERK1/2 phosphorylation, NF-κB activation, and cell growth (Additional file 14: Figure S13B–13D). These results indicated that PI3K-controlled PAK1 does not contribute to ILK expression. HSP90 is a chaperone that associates with client proteins [62-65]. HSP90 regulates ILK stability [25] through an interaction at amino acids 377–406 within the kinase domain of ILK. HSP90 can maintain the tumor-like character of rheumatoid synovial cells by stabilizing ILK, ERK, and AKT [16]. However, the potential regulation of ILK/ERK/AKT signaling by HSP90 remains undetermined. Our study further demonstrated that the HSP90-associated E3 ligase CHIP was required for ILK stabilization, whereas inhibiting PI3K and HSP90 caused CHIP-proteasome-mediated ILK degradation. Radovanac et al. demonstrated that ILK stability is negatively regulated by CHIP ubiquitination in fibroblasts when HSP90 is inhibited [26]. The molecular stabilization of ILK by HSP90 and CHIP remains unclear, but HSP90 interacts with the kinase domain of ILK [25,26], and CHIP-mediated ubiquitylation occurs at the ANK repeats of ILK [26]. CHIP regulates ILK stability in conjunction with others, and this regulation is important for ILK-regulated ERK1/2 and NF-κB activation and cell growth. Regarding the multiple oncogenic effects of ILK, the results of this study and those of previous studies reveal the molecular mechanism of ILK stabilization and expression; furthermore, we showed that ILK promotes cancer cell growth, migration, and survival responses through ERK1/2-regulated NF-κB activation.

The limitation of this study is that the changes observed were in the AGS cells, which have *PIK3CA* and *KRAS* mutations [37] and decreased PTEN expression. To confirm the finding that ILK facilitates ERK1/2 activation in response to physiological and pathological stimuli, we showed that hydrogen peroxide and *H. pylori* infection caused ILK-regulated ERK1/2 activation. In addition, the exogenous administration of EGF confirmed the requirement of ILK and IQGAP1 in EGF-induced ERK1/2 activation, NF-κB activation, cell growth, and migration [47,48]. Crosstalk between EGFR and integrins facilitates gastric cancer cell invasion and proliferation [66]. Moreover, the overexpression of WT EGFR and EGFR mutated at domain VIII may induce PI3K/HSP90-regulated ILK stabilization followed by ERK1/2 activation [48,52]. Regarding the critical role of Ras/ERK1/2 in cell growth advantages [27], the aforementioned results elucidate the significance of ILK in cell growth advantages, at least in gastric cancers, through a non-canonical mechanism involving the facilitation of ERK1/2 activation.

## Conclusion

Based on the finding that PI3K/PTEN/HSP90-regulated ILK upregulation induces non-canonical IQGAP1/Ras/ERK1/2-mediated NF-κB activation and growth advantages in gastric cancer cells, targeting this pathway may be beneficial when used in combination with other anticancer agents. Our hypothesis requires further in vivo investigation with an appropriate animal model.

## Materials

### Chemicals and antibodies

The ILK inhibitor T315 was a gift from Dr. Ching-Shih Chen, College of Pharmacy, The Ohio State University [36]. The NF-κB inhibitor caffeic acid phenethyl ester (CAPE); c-Raf inhibitor GW5074; MEK inhibitors U0126 and PD98059; PI3K inhibitor 2-(4-morpholinyl)-8-phenyl-4H-1-benzopyran-4-one hydrochloride (LY294002); translation inhibitor cycloheximide; proteasome inhibitor MG132; Ras inhibitor FTI-277; HSP90 inhibitor 17-AAG; 5-fluorouracil (5-FU); cisplatin; dimethyl sulfoxide (DMSO); 4,6-diamidino-2-phenylindole (DAPI); and propidium iodide (PI) were purchased from Sigma–Aldrich (St. Louis, MO, USA). Antibodies against phosphorylated AKT at Ser473 (pAKT S), phosphorylated AKT at Thr308 (pAKT T), AKT, phosphorylated GSK-3α/β at Ser21/Ser9 (pGSK-3α/β), GSK-3α/β, phosphorylated PDK1 at Ser241 (pPDK1), PDK1, phosphorylated PAK1 at Thr423 (pPAK1), PAK1, phosphorylated c-Raf at Ser338 (pc-Raf), c-Raf, phosphorylated MEK1/2 at Ser217/Ser221 (pMEK1/2), MEK1/2, phosphorylated ERK1/2 at Thr202/Tyr204 (pERK1/2), ERK1/2, phosphorylated p90RSK at Thr573 (pRSK T), phosphorylated p90RSK at Thr359/Ser363 (pRSK T/S), phosphorylated p90RSK at Ser380 (pRSK S), RSK, phosphorylated IκBα at Ser32 (pIκBα), IκBα, NF-κB, PTEN, HSP90, CHIP, cullin-4A/4B, ubiquitin, and anti-EGFP were purchased from Cell Signaling Technology (Beverly, MA, USA). Anti-ILK, anti-phosphorylated NF-κB at Ser536 (pNF-κB), anti-Ras, and anti-IQGAP1, anti-IQGAP2, and anti-IQGAP3 antibodies were purchased from Santa Cruz Biotechnology (Santa Cruz, CA, USA). Antibodies against Ki-67 and NF-κB were purchased from Abcam (Cambridge, MA, USA). Mouse monoclonal antibodies specific for β-actin and FLAG were purchased from Chemicon International (Temecula, CA, USA). Alexa Fluor 488- and horseradish peroxidase (HRP)-conjugated goat anti-mouse, goat anti-rabbit, and donkey anti-goat IgG antibodies were purchased from Invitrogen (Carlsbad, CA, USA). Cytotoxicity assays were performed to assess drug toxicities before the experiments; non-cytotoxic doses were used in this study.

### Gastric cancer specimens

In this study, 150 cores, including reactive and cancerous (different grades and stages) tissues of the stomach, were obtained from a commercial tissue microarray (BioChain Institute, Inc., Hayward, CA, USA) built for immunohistochemical interrogation. AGS-derived gastric nodules in BALB/c mice were accordingly arrayed [67]. For animal studies, 6- to 8-week-old male wild-type BALB/c mice were purchased from Jackson Laboratory (Bar Harbor, ME, USA) and fed standard laboratory food in the Laboratory Animal Center of National Cheng Kung University. The animals were raised and handled according to the guidelines established by the National Science Council, Taiwan. The experimental protocols adhered to the rules of the Animal Protection Act of Taiwan and were approved by the Laboratory Animal Care and Use Committee of National Cheng Kung University.

### Cell lines and cell culture

AGS (CRL-1739, ATCC; derived from a biopsy specimen of an untreated human gastric adenocarcinoma harboring *KRAS*, *PIK3CA*, *CDH1*, and *CTNNB1* mutations), metastatic MKN45 (JCRB0254, The RIKEN Cell Bank, Japan; established from a poorly differentiated adenocarcinoma of the medullary type from the stomach of a 62-year-old woman bearing an E-cadherin mutation), and SNU-1 human gastric adenocarcinoma cells (CRL-5971, ATCC; derived from a metastatic ascites site of a poorly differentiated primary stomach carcinoma harboring *KRAS* and *MLH1* mutations), and GES-1 gastric epithelial immortalized cells, kindly provided by Dr. Pei-Jung Lu, National Cheng Kung University, were routinely grown in plastic cell culture dishes in Ham’s F-12 nutrient mixture, DMEM, or RPMI 1640 (F-12, RPMI; Invitrogen Life Technologies, Rockville, MD, USA) with l-glutamine and 15 mM HEPES supplemented with 10% heat-inactivated fetal bovine serum (FBS; Invitrogen Life Technologies), 50 units of penicillin, and 50 μg/mL of streptomycin and maintained in a humidified atmosphere with 5% CO_2_ and 95% air.

### Immunohistochemical/immunocytochemical staining

Tissue blocks were fixed overnight at 4°C with 4% neutral buffered paraformaldehyde solution, dehydrated, cleared with HistoClear II (National Diagnostics, Atlanta, GA, USA), and embedded in wax. For immunohistochemical staining, tissue sections were deparaffinized, rehydrated, incubated with 3% H_2_O_2_ in methanol for 15 min, and subjected to heat-induced antigen retrieval by boiling for 10 min in 0.01 M citric acid. For immunocytochemical staining, cells were fixed in 3.7% formaldehyde in PBS for 10 min. After washing twice with PBS, the tissue sections and cells were mixed with primary antibodies in antibody diluents (Dako Corporation, Carpinteria, CA, USA) and incubated overnight at 4°C. The following day, samples were washed with PBS and incubated with or without HRP- or fluorescence-labeled secondary antibodies at room temperature for 1 h. For immunohistochemistry, antibodies against ILK, Ki-67, pNF-κB, NF-κB, and pERK1/2 were used. HRP-reactive sections were washed with PBS, developed with an AEC substrate, counterstained with hematoxylin, and visualized using a microscope (IX71; Olympus, Tokyo, Japan). For confocal microscopy, DAPI (5 μg/mL) was used for nuclear staining. The sections were then visualized using a confocal laser scanning microscope (Digital Eclipse C1si-ready; Nikon, Tokyo, Japan). We alternatively used the TissueFAXS system (TissueGnostics, Vienna, Austria) to analyze the immunostaining of the tissues. In HistoQuest software (TissueGnostics, Tarzana, CA, USA), we plotted the X and Y axes for the expression of the indicated proteins. Therefore, the upper-right region of dot-plot shows double-positive expression but not coexpression. The stained tissue microarray slides were digitized using the TissueFAXS system. HistoQuest software was used for detecting and quantifying stained regions. Two markers, the AEC master marker (ILK, pNF-κB, or Ki-67) and the hematoxylin non-master marker, were analyzed and calculated.

### Cell viability and cytotoxicity assays

To measure cell growth, cell viability was determined using a colorimetric assay (Cell Counting Kit-8; Dojindo Molecular Technologies, Kumamoto, Japan) according to the manufacturer’s instructions. A microplate reader (SpectraMax 340PC; Molecular Devices Corporation, Sunnyvale, CA, USA) was used to measure the absorbance at 450 nm, and the data were analyzed using Softmax Pro software (Molecular Devices Corporation). The relative growth rate was calculated by normalization to the control group. To evaluate cell damage, lactate dehydrogenase (LDH) activity was determined using a colorimetric assay (Cytotoxicity Detection Kit; Roche Diagnostics, Lewes, UK) according to the manufacturer’s instructions. Aliquots of culture media were transferred to 96-well microplates. SpectraMax 340PC was used to measure the absorbance at 620 nm with a reference wavelength of 450 nm, and the data were analyzed using Softmax Pro software.

### Western blotting

Harvested cells were lysed in a buffer containing 1% Triton X-100, 50 mM Tris (pH 7.5), 10 mM EDTA, 0.02% NaN_3_, and a protease inhibitor cocktail (Roche Boehringer Mannheim Diagnostics, Mannheim, Germany). After a freeze–thaw cycle, cell lysates were centrifuged at 10,000 × *g* at 4°C for 20 min. The lysates were boiled in a sample buffer for 5 min. Proteins were then subjected to SDS-PAGE and transferred to PVDF membranes (Millipore, Billerica, MA, USA) using a semi-dry electroblotting system. After blocking with 5% skim milk in PBS, the membranes were incubated overnight with a 1:1,000 dilution of primary antibodies at 4°C. The membranes were then washed with 0.05% PBS-Tween 20 and incubated with a 1:5,000 dilution of HRP-conjugated secondary antibody at room temperature for 1 h. After washing, the membranes were soaked in ECL solution (PerkinElmer Life and Analytical Sciences, Inc., Boston, MA, USA) for 1 min and exposed to an X-ray film (BioMax; Eastman Kodak, Rochester, NY, USA). The relative signal intensity was quantified using ImageJ software (version 1.41o; W. Rasband, National Institutes of Health, Bethesda, MD, USA). The changes in the ratio of proteins compared with the normalized value of untreated cells (indicated protein/β-actin or phosphorylated protein/total protein/β-actin) are also determined. One set of representative data obtained from three independent experiments is shown and the data shown as the mean ± SD values from three independent experiments (Additional file 15: Figure S14).

### Lentiviral-based RNAi transfection

Protein expression was downregulated using lentiviral-based short hairpin RNA (shRNA) targeting the indicated sequences of the different genes, as summarized in Additional file 16: Table S1. Luciferase shRNA (shLuc) was used as a negative control. shRNA clones were obtained from the National RNAi Core Facility, Institute of Molecular Biology and Genomic Research Center, Academia Sinica, Taipei, Taiwan. Lentiviruses were produced by the RNAi Core Facility, National Cheng Kung University. Cells were transduced by lentiviruses with an appropriate multiplicity of infection in complete growth medium supplemented with polybrene (Sigma–Aldrich). After transduction for 24 h and puromycin (Calbiochem, San Diego, CA, USA) selection for 3 days, protein expression was monitored by western blotting. IQGAP1 expression was silenced using the commercial siRNA IQGAP1-HSS113014, containing the following siRNA target sequences: 5′-UUUAGCUGCAGGAAUCUGUAGGGCC-3′ and 5′-GGCCCUACAGAUUCCUGCAGCUAAA-3′ (Invitrogen). Transfection was performed by electroporation by using a pipette-type microporator (Microporator system; Digital Bio Technology, Suwon, Korea). After transfection, cells were incubated for 18 h in RPMI 1640 at 37°C before infection. A nonspecific scrambled siRNA (Stealth RNAi^™^ siRNA Negative Control Kit, 12935–100; Invitrogen) was used as the negative control.

### Electrophoretic mobility shift assays

Cells were incubated in 300 μL buffer A [10 mM HEPES (pH 7.9), 1.5 mM magnesium chloride, 10 mM potassium chloride, 0.5 mM phenylmethylsulfonyl fluoride, 0.5 mM dithiothreitol, 2 μg/mL leupeptin, 10 μg/mL aprotinin, 50 mM sodium fluoride, and 1 mM sodium orthovanadate] on ice for 10 min and gently shaken for 10 s. The pellet containing crude nuclei was collected using centrifugation at 12,000 × *g* for 10 s, resuspended in 30 μl buffer C [20 mM HEPES (pH 7.9), 25% glycerol, 420 mM sodium chloride, 1.5 mM magnesium chloride, 0.2 mM EDTA, 0.5 mM phenylmethylsulfonyl fluoride, 0.5 mM dithiothreitol, 2 μg/mL leupeptin, 10 μg/mL aprotinin, 50 mM sodium fluoride, and 1 mM sodium orthovanadate] by vortexing for 15 s, followed by incubation on ice for 20 min. After centrifuging the cells at 12,000 × *g* for 2 min, the supernatant containing nuclear proteins was collected, quantified (BCA Protein Assay Reagent; Pierce Biotechnology, Inc., Rockford, IL, USA), and stored in aliquots at −70°C. The electrophoretic mobility shift assay (EMSA) used the following oligonucleotides as probes: NF-κB (f): 5′-CAA ATG TGG GAT TTT CCC ATG AGT; NF-κB (r): 5′-GAC TCA TGG GAA AAT CCC ACA TTT G. The forward and reverse oligonucleotides (30 pmol) were placed in 23 μL of DNA polymerase buffer (Klenow 1X; Promega, Madison, WI, USA), heated at 94°C for 2 min, and annealed at room temperature for 30 min. The annealed double-stranded oligonucleotides were end-labeled using a fill-in reaction with Klenow polymerase. One unit of Klenow and 40 μCi of [^32^P] dCTP (PerkinElmer Life and Analytical Sciences, Inc.) were added to the annealed oligonucleotides, and the mixture was incubated at 30°C for 15 min. The labeled oligonucleotides were purified using G-50 columns (Sephadex; PerkinElmer Life and Analytical Sciences, Inc.). The DNA-binding reaction was performed at 4°C for 30 min in a mixture containing 3 μg of nuclear extract, 10 mM Tris–HCl [pH 7.5], 50 mM sodium chloride, 0.5 mM dithiothreitol, 0.5 mM EDTA, 1 mM magnesium chloride, 4% glycerol, 0.05 μg poly (dI-dC)-poly (dI-dC) (PerkinElmer Life and Analytical Sciences, Inc.), and 2 × 10^4^ cpm ^32^P-labeled double-stranded oligonucleotides. Samples were analyzed on a 4% polyacrylamide gel (acrylamide/bis-acrylamide 29:1 in 0.5× Tris-borate–EDTA buffer) at 10 V/cm for 2 h. The gel was dried and analyzed using a quantitative autoradiography densitometer.

### Luciferase reporter assay

To analyze NF-κB promoter activity by a luciferase reporter assay, transient transfection was performed using the GeneJammer transfection reagent (Stratagene, La Jolla, CA, USA). In short, cells were cotransfected with 0.2 μg of an NF-κB-promoter-driven firefly luciferase reporter and 0.01 μg of a Renilla luciferase-expressing plasmid (pRL-TK; Promega). Twenty hours after transfection, the cells were lysed and harvested for firefly and Renilla luciferase using the Dual-Glo luciferase assay system (Promega). For each lysate, firefly luciferase activity was normalized to Renilla luciferase activity to assess transfection efficiencies.

### Ras pull-down assay

Ras activation assays were performed according to the affinity precipitation protocol provided by the manufacturer (Ras Activation Assay Kit 28820; Single Oak Drive, Temecula, CA, USA). Cell lysates were incubated with Raf-1 RBD for 45 min at 4°C and centrifuged to pellet agarose beads. The agarose beads were washed, and the pellets were resuspended in 2× Laemmli sample buffer and boiled for 5 min. The supernatant was collected, and cellular proteins were resolved by SDS-PAGE and analyzed by immunoblotting.

### Coimmunoprecipitation

For coimmunoprecipitation, 100 μg cell lysate from cells was incubated overnight at 4°C with 5 μg protein G (Amersham Biosciences, Uppsala, Sweden) and 2 μg of antibodies. The expression of interacting proteins was determined by Western blotting.

### Transfection

Transient transfection was performed using an MP-100 Microporator (Digital Biotechnology) according to the manufacturer’s instructions for optimization and usage. The plasmid expressing EGFP-PTEN (ID NM_000314; Plasmid 13039) and its control pcDNA3-EGFP (Plasmid 13031); pWZL-Neo-Myr-Flag-ILK (ID NM 004517; Plasmid 20505) and its control pWZL-Neo-Myr-Flag-Dest (Plasmid 15300); and wild-type EGFR (Plasmid 11011), EGFR dominant-negative mutant (EGFRVIII) (Plasmid 11015) and their control vector pBABE-puro (Plasmid 1764) were purchased from Addgene (Cambridge, MA, USA). ILK dominant-negative mutant (ILK_A262V_) (ID NM_004517) was purchased from OriGene Technologies, Inc. (Rockville, MD, USA). Three FLAG-tagged recombinant ILK proteins, full-length ILK_1–452_, N-terminal truncated ILK_171–452_, and C-terminal truncated ILK_1–171_, were constructed as described previously [18]. After transfection, the cells were cultured for 24 h before the experiments.

### PI3K activity assay

A PIP3 Mass ELISA (K-2500 s, Echelon Biosciences, Salt Lake City, UT, USA) was performed to detect PI3K activity in cells according to the manufacturer’s instructions.

### Wound healing assay

For cell migration assays, the confluent monolayers of cells were wounded by scraping a pipette tip across the monolayer. The cells were washed with PBS and incubated with appropriate media. Images at 100× magnification were taken at wounding and 12 h later by using a microscope (IX71; Olympus). Cell migration was assessed using ImageJ software and was reported as the number of cells that migrated into the scraped region.

### Apoptosis assay

Apoptosis was assessed using nuclear propidium iodide (PI; Sigma–Aldrich) staining and flow cytometry (FACSCalibur; Becton Dickinson, San Jose, CA, USA) with excitation at 488 nm and emission in the FL2 channel (565–610 nm). Samples were analyzed using CellQuest Pro 4.0.2 software (Becton Dickinson), and quantification was performed using WinMDI 2.8 software (The Scripps Institute, La Jolla, CA, USA). Apoptosis levels were reported as the percentages of sub-G_1_ phase cells.

### Statistical analyses

Values were expressed as mean ± standard deviation (SD). Significant differences between groups were assessed using one-way ANOVA followed by Dunnett’s post hoc test as appropriate, Student’s *t* test, or analysis of variance. Analyses were performed using GraphPad Prism 4 software (GraphPad Software Inc., La Jolla, CA, USA). After the densities of the detected proteins were quantified using HistoQuest software, logistic regression analysis of the graded expression of ILK, phosphorylated NF-κB Ser536, and Ki-67 in 93 gastric cancer specimens tested was performed. After the AEC-based immunohistochemistry assay, no AEC staining was considered negative, <25% staining was considered grade 1, between 25% and 50% staining was considered grade 2, and >50% staining was considered grade 3. Data analysis was performed using multinomial logistic regression and the pseudo R-squared test in GraphPad Prism 4 software (GraphPad Software Inc.). The exact *P*-values are listed in the corresponding figure legends. Statistical significance was set at *P* <0.05.

### Supplemental materials and methods

The additional information was also attached (Additional file 1: Supplemental materials and methods).

## Additional files

Additional file 1:
**Supplemental materials and methods.**
Additional file 2: Figure S1. Expression of ILK in gastric tumors. The representative AEC-based immunohistochemical staining of ILK and Ki-67 in 2 gastric tumor patients. Hematoxylin is used for nuclear counterstaining. Additional file 3: Figure S2. ILK regulates cell growth and NE-κB activation. **(A)** WST-8-based assay showed cell growth hi gastric cancer cell lines AGS, SNU-l, MKN45, and GES-l compared with the status at Day 0. Western blotting **(B)** and immunostaining **(C)** showed ILK expression. β-actin was used as an internal control. The mean fluorescent intensity (MEl) per tested cell is also shown. **(B)** Western blotting showed ILK expression in shLuc- and shILK-transfected A549 and Hl975 human lung adenocarcinoma cells, HK2 human renal proximal tubular epithelial cells, and THP 1 human acute monocytic leukemia cells. β-actin was used as an internal control. **(E)** Silencing ILK retarded cell growth. **(F)** shLuc- or shILK-transfected AGS cells were cultured for 14 days, and colony formation was manually counted. PI staining followed by flow cytometry analysis showed the cell cycle in shLuc- or shILK-transfected AGS cells treated with vinorelbine (VNR. 1 μM) **(G)** and in AGS cells treated with T315 **(H)** for 24 h compared with DMSO-treated groups. The percentages of cells at the indicated phases are shown. **(I)** Immunostaining showing the nuclear translocation of NF-κB. **(J)** NF-κB activation in shLuc- and shILK-transfected cells was determined. For cell growth, cell cycle, and luciferase activity, data are the means ± SD from three individual experiments. tp < 0.001 compared with Day 0 or relative control. #P <0.05 and ##P < 0.01 compared with shLuc. Additional file 4: Figure S3. Expression of ILK, phosphorylated NF-κB, and Ki-67 in. gastric tumors. **(A)** The representative AEC-based immunohistochemical staining showed the expression of ILK, phosphorylated NF-κB at Ser536, and Ki-67 in a gastric tissue array containing 150 cases. Hematoxylin is used for nuclear counterstaining. An example of triple-positive staining is shown. **(B)** Ninety-three of 150 cases of gastric cancer specimens were statistically analyzed for the graded expression (Grade I, <25%; Grade II, 25-50%; Grade III, >50% of observed region) of these proteins by a logistic regression analysis. Additional file 5: Figure S4. ILK facilitates ERK1/2 signaling. **(A)** The Human Phospho-Kinase Array (Catalog # ARY003B, R & D systems) was performed to detect the phosphorylation of 43 kinases and 2 related total proteins in AGS and MKN45 cells. The mean raw density is shown as the mean data of changed kinases and the positive controls as indicated. Western blotting showed the expression of the indicated proteins in shLuc- and shILK-transfected cells **(B)**, and PD98059-treated AGS cells **(C)**, and β-actin was used as an internal control. **(D)** Cell cycle analysis showed the effects of PD98059 treatment for 48 h. **(E)** Western blotting showed the expression of IQGAP1, IQGAP2, and IQGAP3 in AGS, MKN45, and shLuc- or shILK-transfected AGS cells. In shLuc- or shIQGAP3-transfected AGS cells, the expression of the indicated proteins **(F)**, NF-κB activation **(G)**, and cell growth **(H)** were also determined. For cell cycle, luciferase activity, and proliferation assay, data are the means ± SD from three individual experiments. NS, not significant. Regular triangle, increased expression; inverted triangle, decreased expression. Additional file 6: Figure S5. Expression of ILK, IQGAP1, and Ras in AGS cells. The representative fluorescence-based immunohistochemical staining of ILK (*green*), IQGAP1 (*red*), and Ras (*red*) in AGS cells. DAPI (*blue*) is used for nuclear counterstaining. Additional file 7: Figure S6. Expression of integrins in AGS and MKN45 cells. One representative histogram of immunostaining followed by flow cytometry analysis shows the expression of β1 and β3 integrins and the active form of β1 integrin in AGS and MKN45 cells. 2°Ab, secondary antibody. Additional file 8: Figure S7. ILK facilitates ERK1/2 phosphorylation. AGS cells were treated with PD98059, LY294002, T315, or Ras inhibitor FTI-277 for the indicated times. Western blotting showed the expression of the indicated proteins. β-actin was used as an internal control. Additional file 9: Figure S8. AKT is not required for ILK stabilization in AGS cells. Western blotting showed the expression of ILK and phosphorylated AKT at Ser473 (*pAKT S*) in AGS cells treated with LY294002 and AKT inhibitor 5-(2-Benzothiazolyl)-3-ethyl-2-[2-(methylphenylamino) ethenyl]-1-phenyl-1H-benzimidazoli um iodide (*AKTi*, 2.5 μM). β-actin was used as an internal control. Inverted triangle, decreased expression. Additional file 10: Figure S9. HSP90 signaling regulates ILK stabilization and ERK1/2/NF-κB signaling. Western blotting shows the expression of the indicated proteins in AGS cells treated with 17-AAG **(A)** and SAHA in the presence of MG132 **(C)**. β-actin was used as an internal control. **(B)** Reporter assay detected NF-κB activation in AGS cells treated with SAHA (5 μM) for 24 h. Data are the means ± SD from three individual experiments. **P* < 0.05 compared with DMSO. Regular triangle, increased expression; inverted triangle, decreased expression. Additional file 11: Figure S10. MG132 treatment reverses ILK expression to facilitate ERK1/2/NFκB activation in MKN45 cells. Western blotting showed ILK expression and phosphorylation of MEK1/2 and ERK1/2 in MKN45 cells treated with MG132. β-actin was used as an internal control. Regular triangle, increased expression. Additional file 12: Figure S11. Signaling of PI3K/HSP90/CHIP/ILK/ERK1/2/NF-κB determines cell migration. Migration activity was tested in AGS, MKN45, shILK- or siIQGAP1-transfected AGS cells **(A)**, LY294002 (25 μM), 17-AAG (0.2 μM), T315 (2.5 μM), 9 PD98059 (50 μM), or CAPE (25 μg/ml)-treated AGS cells **(B)**, and shLuc- or shCHIP-transfected AGS cells treated with LY294002 (25 μM) and 17-AAG (0.2 μM) **(C)** for 12 h. shLuc, control siRNA, or DMSO were used as the negative controls. A representative image in the observed field is shown. The numbers of migrated cells in the observed field are shown. **(D)** PI staining followed by flow cytometry analysis showed 5-FU- or cisplatin-induced apoptosis for 2 days in AGS or MKN45 cells pre-treated with T315 for 0.5 h. DMSO were used as the negative controls. Data are the means ± SD from three individual experiments. **P* < 0.05, ***P* < 0.01 and ****P* < 0.001 compared with relative control. #*P* < 0.05, ##*P* < 0.01, and ###*P* < 0.001 compared with relative control. Additional file 13: Figure S12. ILK individually determines activation of AKT and ERK1/2 in response to different stimuli. Western blotting showed the phosphorylation of AKT at Ser473 (*pAKT* S) and ERK1/2 at Tyr202/Thr204 (*pERK*1/2) in shLuc- or shILK-transfected A549 or MKN45 cells with EGF (10 ng/ml) **(A)** and hydrogen peroxide (*H2O2*, 10 μM) **(B)** treatment for 1 h or *Helicobater pylori* (*H. pylori*, MOI = 10) infection for 6 h **(C)**. β-actin was used as an internal control. Regular triangle, increased expression; inverted triangle, decreased expression. Additional file 14: Figure S13. PAK1 is regulated for c-Raf/ERK1/2 signaling, NF-κB activation and cell proliferation but not for ILK stabilization. AGS cells were treated with LY294002 for 24 h **(A)** and transfected with shLuc and shPAK1 clones #1 and #2 **(B)**. Western blotting showed the expression of the indicated proteins. β-actin was used as an internal control. The activation of NF-κB **(C)** and cell proliferation **(D)** in shLuc- and shPAK1-transfected AGS cells were determined. For luciferase activity and cell proliferation, data are the means ± SD from three individual experiments. ***P* < 0.01 compared with shLuc. Inverted triangle, decreased expression. Additional file 15: Figure S14. Quantification of protein expression. For the immunoblots 10 and immunoprecipitation data in the indicated figures, the relative signal intensity was quantified using ImageJ software. The changes in the ratio of proteins compared with the normalized value of untreated cells (indicated protein/β-actin or phosphorylated protein/total protein/β-actin) are also determined. The data shown as the mean ± SD values from three independent experiments. **P* < 0.05, ***P* < 0.01, and ****P* < 0.001 compared with the relative controls. #*P* < 0.05, ##*P* < 0.01, and ###*P* < 0.001 compared with the indicated controls. ns, not significant. Additional file 16: Table S1. Target sequences of shRNAs used in this study.

## Abbreviations

ANK: Ankyrin
CHIP: HSP90-associated E3 ubiquitin ligase C-terminus of heat shock cognate 70-interacting protein
ECM: Extracellular matrix
ERK: Extracellular signal-regulated protein kinase
GSK: Glycogen synthase kinase
HSP: Heat shock protein
ILK: Integrin-linked kinase
IQGAP: IQ motif-containing GTPase-activating protein
MAPK: Mitogen-activated protein kinase
PH: Pleckstrin homology
PI3K: Phosphatidylinositol 3-kinase
PIP2: Phosphatidylinositol 4,5-bisphosphate (PIP2)
PIP3: Phosphatidylinositol 3,4,5-triphosphate (PIP3)
PTEN: Phosphatase and tensin homolog deleted on chromosome 10
siRNA: Short interfering RNA
shRNA: Short hairpin RNA
