# Supplementary material for: An increase in integrin-linked kinase non-canonically confers NF-κB-mediated growth advantages to gastric cancer cells by activating ERK1/2
Source: Cell Commun Signal. 2014 Nov 15;12:69. doi: 10.1186/s12964-014-0069-3 (PMC4255431; doi:10.1186/s12964-014-0069-3)
Supplement: Additional file 1: — Supplemental materials and methods. [file 12964_2014_69_MOESM1_ESM.doc]

**Supplemental Information**

**Supplemental materials and methods**

**Chemicals and antibodies**

Vinorelbine (VNR), histone deacetylase inhibitor suberoylanilide hydroxamic acid (SAHA), hydrogen peroxide (H2O2), and Akt inhibitor Akti-1/2 were purchased from Sigma-Aldrich (St. Louis, MO). Anti-β1 and β3 intergrins and its active form antibodies were obtained from Abcam (Cambridge, MA). Recombinant human EGF was purchased from PeproTech (Rocky Hill, NJ).

**Cell lines and cell culture**

Human lung adenocarcinoma A549 (CCL185, ATCC) and H1975 (CRL5908, ATCC) cells and human renal proximal tubular epithelial cell line HK-2 cells (CRL-2190, ATCC) were routinely grown on plastic in Dulbecco's modified Eagle's medium (DMEM, Gibco-BRL; Grand Island, NY) with L-glutamine and 15 mM HEPES, supplemented with 10% fetal bovine serum (FBS, Gibco-BRL), 100 units of penicillin, and 100 μg/ml streptomycin and maintained at 37°C in 5% CO2. Human monocytic THP-1 (TIB-202, ATCC) cells were routinely grown on plastic in RPMI Medium 1640 (RPMI; Invitrogen Life Technologies, Rockville, MD), with L-glutamine and supplemented with 10% heat-inactivated FBS (Invitrogen Life Technologies), 50 units of penicillin, and 50 μg/ml of streptomycin and maintained in a humidified atmosphere with 5% CO2 and 95% air.

**Colony forming assay**

Drug-treated cells were seeded in 6-well plates (1500 cells per well) with cytokine-free Methocult H4230 methylcellulose medium (Stem Cell Technologies, Vancouver, BC, Canada) and cultured at 37°C in 5% CO2 and ≥ 95% humidity for 7 days. The number of colonies was 2 counted and manually scored after the addition of 1 mg/ml 3-(4,5-dimethylthiazol-2-yl)-2,5-diphenyltetrazolium bromide.

**Bacterial strains and culture condition**

The wild type *Helicobacter pylori* (*H. pylori*) strain ATCC43504 was derived from the American Type Culture Collection (ATCC). *H. pylori* bacteria were grown on CDC anaerobic 5% sheep blood agar plates (BBL, Becton-Dickinson, San Jose, CA) under microaerophilic conditions (5% O2, 10% CO2, 85% N2) and 85% humidity in a Nuaire incubator (Plymonth, MN) at 37°C.

**Immunocytochemical staining**

Cells were then fixed in 3.7% formaldehyde in PBS for 10 min. After washing twice with PBS, tissue sections and cells were mixed with anti-ILK antibodies in antibody diluents (DAKO Corporation, Carpinteria, CA) and incubated at 4°C overnight. The next day, samples were washed with PBS and then incubated with or without fluorescence-labeled secondary antibodies at room temperature for 1 h. For confocal microscopy, DAPI (5 μg/ml) was used for nuclear staining. The cells were then visualized using a confocal laser scanning microscope (Digital Eclipse C1 si-ready; Nikon, Tokyo, Japan). The confocal images were generated using EZ-C1 software (Nikon). For quantifying the level of ILK, we used the same protocol to establish the standard RGB intensity range and then used the mean fluorescent intensity (MFI) as the level of ILK. For flow cytometric analysis, cells were stained with anti-β1 and β3 intergrins and its active form antibodies and then incubated with a mixture of Alexa Fluor 488-conjugated goat anti-rabbit IgG. Cells were analyzed using flow cytometry (FACSCalibur; BD Biosciences, San Jose, CA) with excitation set at 488 nm; emission was detected with the FL-1 channel (515-545 nm). Samples were analyzed using CellQuest Pro 4.0.2 software (BD Biosciences). Small cell debris was excluded by gating on a forward scatter plot.

**Cell cycle assay**

The cell cycle was analyzed using nuclear propidium iodide (PI; Sigma-Aldrich) staining and then analyzed using flow cytometry (FACSCalibur; Becton Dickinson) with excitation set at 488 nm and emission detected at the FL-2 channel (565-610 nm). The distribution of cells in different phases of the cell cycle was calculated using the MetaMorph software (Molecular Devices, Downingtown, PA).

**Human phospho-kinase array**

The cell lysates were applied and incubated with the human phospho-MAPK Array Kit (R & D Systems, Minneapolis, MN) as per the manufacturer's instructions. The data were quantified by using NIH image software analysis (Universal Imaging Corp., Downingtown, PA).
