# Supplementary material for: An increase in integrin-linked kinase non-canonically confers NF-κB-mediated growth advantages to gastric cancer cells by activating ERK1/2
Source: Cell Commun Signal. 2014 Nov 15;12:69. doi: 10.1186/s12964-014-0069-3 (PMC4255431; doi:10.1186/s12964-014-0069-3)
Supplement: Supplementary file 7 [file 12964_2014_69_MOESM15_ESM.doc]

Tseng *et al.* Figure S14

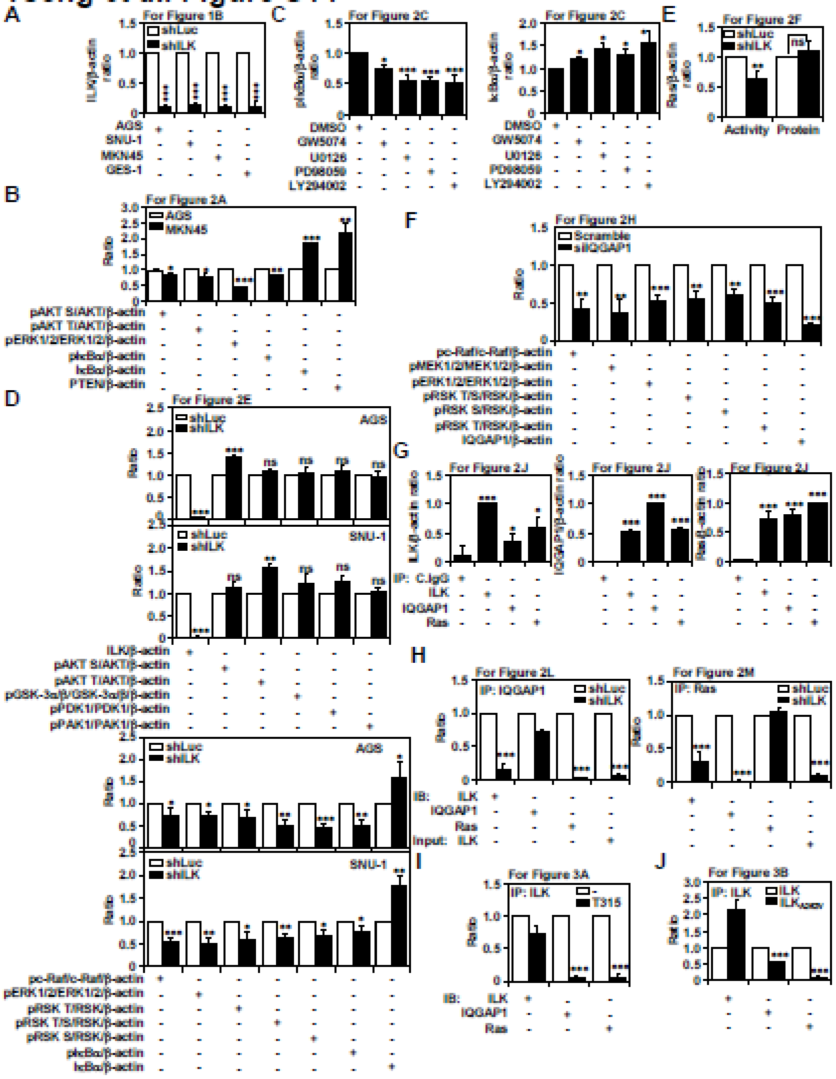

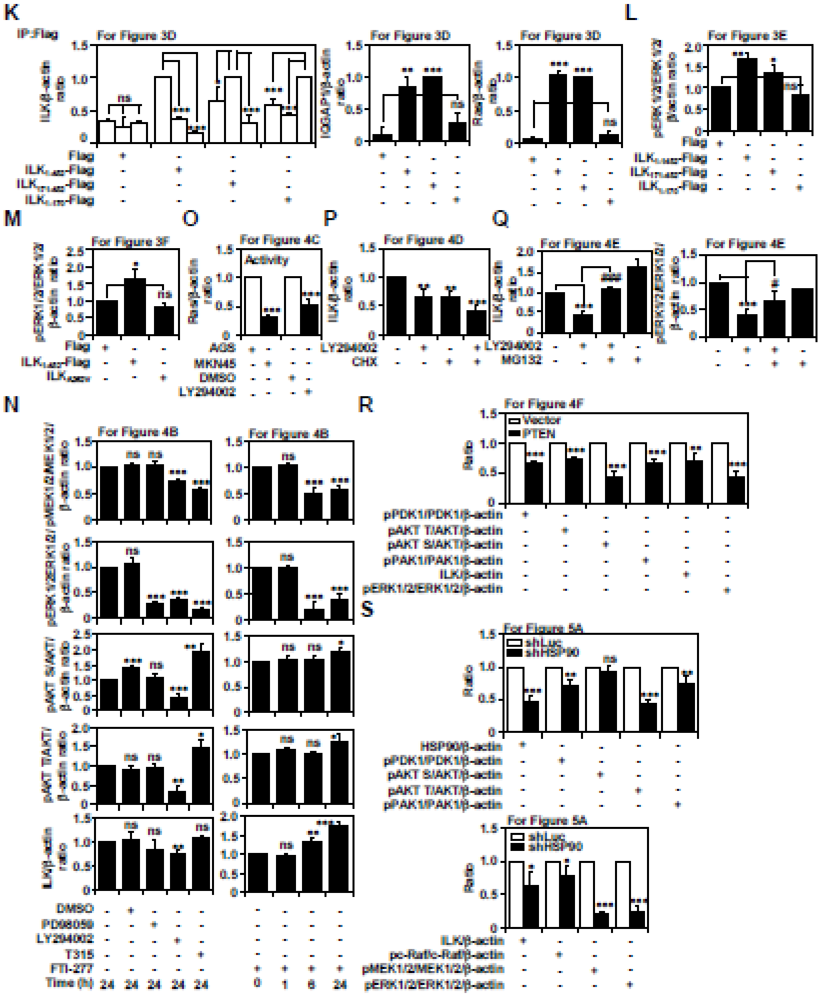

K
IP:Flag
For Figure 3D
ILK/β-actin ratio
IQGAP1/β-actin ratio
Ras/β-actin ratio
Flag
ILK1-452-Flag
ILK171-452-Flag
ILK1-170-Flag
L
For Figure 3E
pERK1/2/ERK1/2/β-actin ratio
M
For Figure 3F
ILKR211A
O
For Figure 4C
Activity
AGS
MKN45
DMSO
LY294002
P
For Figure 4D
CHX
Q
For Figure 4E
MG132
N
For Figure 4B
pMEK1/2/MEK1/2/β-actin ratio
pERK1/2/ERK1/2/β-actin ratio
pAKT S/AKT/β-actin ratio
pAKT T/AKT/β-actin ratio
ILK/β-actin ratio
DMSO
PD98059
LY294002
T315
FTI-277
Time (h)
R
For Figure 4F
Vector
PTEN
Ratio
pPDK1/PDK1/β-actin
pAKT T/AKT/β-actin
pAKT S/AKT/β-actin
pPAK1/PAK1/β-actin
ILK/β-actin
pERK1/2/ERK1/2/β-actin
S
For Figure 5A
shLuc
shHSP90
HSP90/β-actin
pc-Raf/c-Raf/β-actin
pMEK1/2/MEK1/2/β-actin

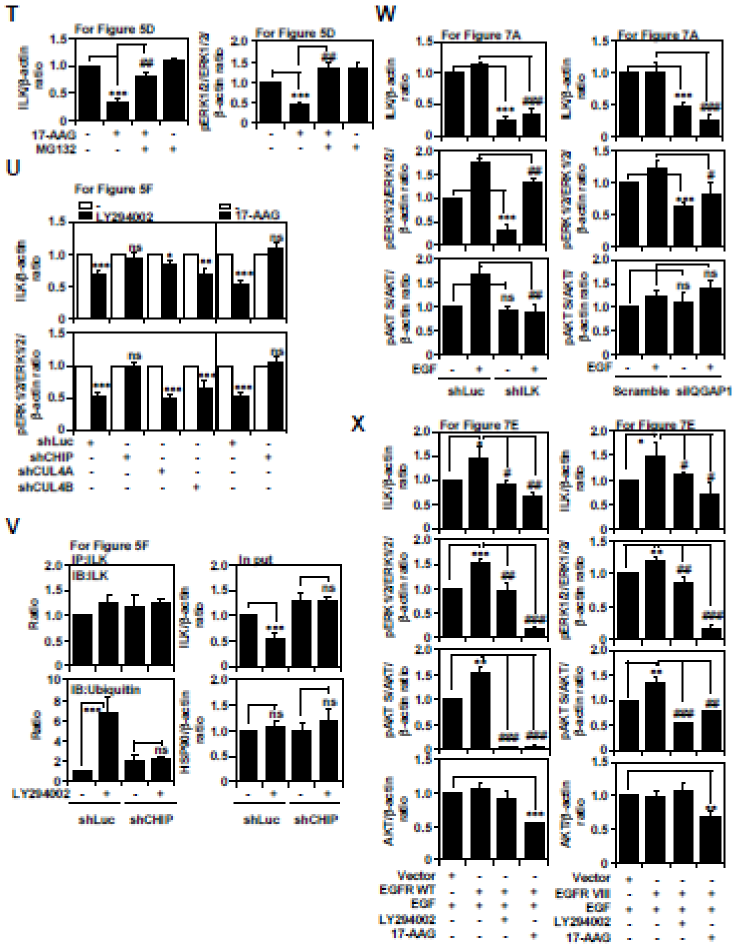
T
For Figure 5D
ILK/β-actin ratio
pERK1/2/ERK1/2/ β-actin ratio
17-AAG
MG132
U
For Figure 5F
LY294002
17-AAG
ILK/β-actin ratio
pERK1/2/ERK1/2/ β-actin ratio
shLuc
shCHIP
shCUL4A
shCUL4B
V
For Figure 5F
IP:ILK
IB:ILK
IB:Ubiquitin
Ratio
In put
ILK/β-actin ratio
HSP90/β-actin ratio
LY294002
shLuc
shCHIP
W
For Figure 7A
ILK/β-actin ratio
pERK1/2/ERK1/2/ β-actin ratio
pAKT S/AKT/ β-actin ratio
EGF
shLuc
shILK
Scramble
siIQGAP1
X
For Figure 7E
ILK/β-actin ratio
pERK1/2/ERK1/2/ β-actin ratio
pAKT S/AKT/ β-actin ratio
AKT/β-actin ratio
Vector
EGFR WT
EGFR VIII
EGF
LY294002
17-AAG
