# Supplementary material for: An increase in integrin-linked kinase non-canonically confers NF-κB-mediated growth advantages to gastric cancer cells by activating ERK1/2
Source: Cell Commun Signal. 2014 Nov 15;12:69. doi: 10.1186/s12964-014-0069-3 (PMC4255431; doi:10.1186/s12964-014-0069-3)
Supplement: Supplementary file 8 [file 12964_2014_69_MOESM16_ESM.doc]

Supplemental Table

Table S1

Target gene	Clone	Sequence	Work in AGS	For this study
CUL4A	1	CTGATAGACAGAGACTATATG	++	
	2	GGACAAGAAGATGTTACTAAA	++	
	3	GCAGAACTGATCGCAAAGCAT	+++	AGS
CUL4B	1	GCCATGAAAGAAGCATTTGAA	+++	AGS
	2	CCTGAATATCTACATCATGTT	++	
HSP90	1	GTTATCCTACACCTGAAAGAA	+++++	AGS
	2	GAAGGATGGTGACAAGAAGAA	++	
	3	TATGGCATGACAACTACTTTA	++++	
	4	TACTTGGAGGAACGAAGAATA	+++	
	5	GTTATCCTACACCTGAAAGAA	++	
IQGAP1	1	GCCAAAGTCAATGTCAACCTT	+++	
	2	GCCCAAGATGACTACAGGATA	++++	AGS
	3	GCCGATATCATACAGTTCCAT	++	
ILK	1	CCGTAGTGTAATGATTGATGA	+++++	AGS, SNU-1, MKN45, GES-1, A549, H1975, HK2, and THP1
	2	TCAGAGCTTTGTCACTTGCCA	+++	
	3	TCAGAGCTTTGTCACTTGCCA	+++++	
	4	GCAATGACATTGTCGTGAAGG	++++	
	5	TCAGAGCTTTGTCACTTGCCA	++++	
Luc	1	ATCACAGAATCGTCGTATGCA	Non specific shRNA	AGS, SNU-1, MKN45, GES-1, A549, H1975, HK2, and THP1
PAK1	1	CCAGAGGTTGTGACACGAAAG	+++++	AGS
	2	GCATTCGAACCAGGTCATTCA	+++	
	3	CCCTAAACCATGGTTCTAAAC	++++	

Supplemental Table

Table S1

**Target gene**	**Clone**	**Sequence**	**Work in AGS**	**For this study**
CUL4A	1	CTGATAGACAGAGACTATATG	++	
	2	GGACAAGAAGATGTTACTAAA	++	
	3	GCAGAACTGATCGCAAAGCAT	+++	AGS
CUL4B	1	GCCATGAAAGAAGCATTTGAA	+++	AGS
	2	CCTGAATATCTACATCATGTT	++	
