# Supplementary material for: An increase in integrin-linked kinase non-canonically confers NF-κB-mediated growth advantages to gastric cancer cells by activating ERK1/2
Source: Cell Commun Signal. 2014 Nov 15;12:69. doi: 10.1186/s12964-014-0069-3 (PMC4255431; doi:10.1186/s12964-014-0069-3)

# Tseng *et al.* Figure S6

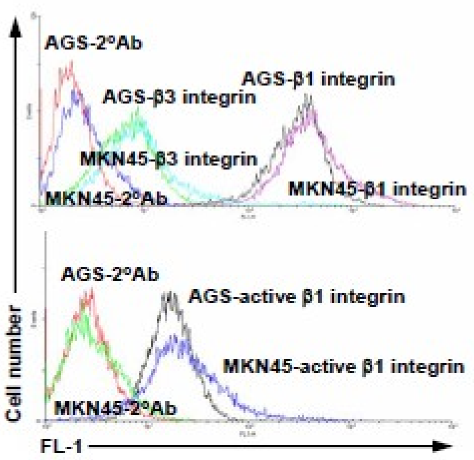

Supplement: Supplementary file 14 [file 12964_2014_69_MOESM7_ESM.doc]
